# Cherry Blossom Forecast Based on Transcriptome of Floral Organs Approaching Blooming in the Flowering Cherry (*Cerasus* × *yedoensis*) Cultivar ‘Somei-Yoshino’

**DOI:** 10.3389/fpls.2022.802203

**Published:** 2022-01-26

**Authors:** Kenta Shirasawa, Tomoya Esumi, Akihiro Itai, Sachiko Isobe

**Affiliations:** ^1^Laboratory of Plant Genetics and Genomics, Department of Frontier Research and Development, Kazusa DNA Research Institute, Kisarazu, Japan; ^2^Laboratory of Pomology and Viticulture, Academic Assembly Institute of Agricultural and Life Sciences, Shimane University, Matsue, Japan; ^3^Laboratory of Plant Resource Science, Department of Agricultural and Life Science, Kyoto Prefectural University, Kyoto, Japan

**Keywords:** cherry blossom forecast, flowering cherry, RNA sequencing, time-course analysis, transcriptomics

## Abstract

To gain insights into the genetic mechanisms underlying blooming and petal movement in flowering cherry (*Cerasus* × *yedoensis*), we performed time-course RNA-seq analysis of the floral buds and open-flowers of the most popular flowering cherry cultivar, ‘Somei-Yoshino.’ Independent biological duplicate samples of floral buds and open-flowers were collected from ‘Somei-Yoshino’ trees grown at three different locations in Japan. RNA-seq reads obtained from floral bud and open-flower samples collected in the current study (in 2019) and in a previous study (in 2017) were aligned against the genome sequence of ‘Somei-Yoshino’ to quantify gene transcript levels. Clustering analysis of RNA-seq reads revealed dynamic changes in the transcriptome, with genes in seven modules predominantly expressed at specific time points, ranging from 5 weeks before flowering to 2 weeks after flowering. Based on the identified gene modules and Gene Ontology (GO) terms enriched at different floral stages, we speculate that the genetic mechanisms underlying petal movement and flower opening in cherry involve the processes of development, cell wall organization, reproduction, and metabolism, which are executed by genes encoding transcription factors, phytohormones, transporters, and polysaccharide metabolic enzymes. Furthermore, we established a statistical model for cherry bloom forecasting, based on gene expression levels as RNA markers at different time points before flowering.

## Introduction

Flowering cherry, also known as sakura, typically blooms in the spring and is valued as a popular ornamental flower across the world. ‘Somei-Yoshino’ (*Cerasus* × *yedoensis*), which is presumed to be an interspecific hybrid between *C. spachiana* and *C. speciosa* (Takenaka, 1963; Innan et al., 1995; Nakamura et al., 2015), is the most popular cultivar of flowering cherry in Japan. Given its genomic heterozygosity and self-incompatibility, ‘Somei-Yoshino’ is propagated by grafting (Iketani et al., 2007). Because of its clonal nature, ‘Somei-Yoshino’ trees planted within a specific location bloom at the same time; however, across the Japanese archipelago, the blooming front progresses from south to north because of differences in environmental conditions. Since the blooming date is important for the tourism industry in the spring season, forecasting methods based on cumulative temperature have been developed to predict the flowering date of cherry blossoms (Aono and Murakami, 2017).

Flowering involves two main processes: floral bud initiation and flower opening. The molecular mechanisms of floral bud initiation have been well studied in *Arabidopsis thaliana* and rice (*Oryza sativa*) as flowering models of long- and short-day plants, respectively, revealing that *FLOWERING LOCUS T* (*FT*) is the key gene involved in floral bud differentiation (Izawa et al., 2003). This mechanism is widely conserved across plant species (Higuchi, 2018). In woody plants, following floral bud initiation, buds enter a period of endo- and eco-dormancy, which are followed by dormancy release and bud break. In the Rosaceae family members, *DORMANCY-ASSOCIATED MADS-box* (*DAM*) genes have been reported to regulate bud dormancy (Yamane, 2014). In our previous study for ‘Somei-Yoshino,’ transcriptome data from the developmental floral bud sample series suggested that endodormancy is almost completed in 2 months before the flower opening (Shirasawa et al., 2019). However, while the physiological aspect of the mechanism for bud break and flower opening following the ecodormancy has been thoroughly investigated, only a few studies have been conducted to explore the genetic basis of this mechanism (van Doorn and Van Meeteren, 2003).

The genome sequence of ‘Somei-Yoshino’ has been determined at the chromosome level, and genes involved in the regulation of dormancy and flowering time have been identified in this cultivar through time-course transcriptome analysis (Shirasawa et al., 2019). Since expression levels of key genes transmit the environmental conditions, such as day-length and temperature, to biological processes such as blooming, the identification of these genes might help predict the blooming date in flowering cherry. Because the transcriptome is affected by environmental conditions (e.g., changes in weather and habitat), biological replications of flowering cherry trees over multiple years and locations would be required for the accurate identification of genes affecting the blooming date.

Whereas interaction between environmental conditions and dormancy have been well characterized in the Rosaceae deciduous trees (Yamane, 2014; Lloret et al., 2017; Tylewicz et al., 2018), those in the process from bud break to flower opening, in post-dormancy stage, has been still unclarified. In this study, we aimed to identify genes uniquely expressed before and during flower opening, and to obtain insights into the molecular mechanisms underlying blooming in flowering cherry. We collected floral buds and open-flowers from ‘Somei-Yoshino’ trees planted at three locations in Japan (Chiba, Kyoto, and Shimane) in 2019, and analyzed their transcriptome using the RNA sequencing (RNA-seq) technology. The time-course transcriptome data generated in the current study and in our previous study (Shirasawa et al., 2019) was used to characterize gene expression patterns in ‘Somei-Yoshino’ floral buds, some of which could be used to forecast the blooming time and to gain insights into the molecular mechanisms controlling blooming in flowering cherry.

## Materials and Methods

### Plant Materials

**FIGURE 1:**
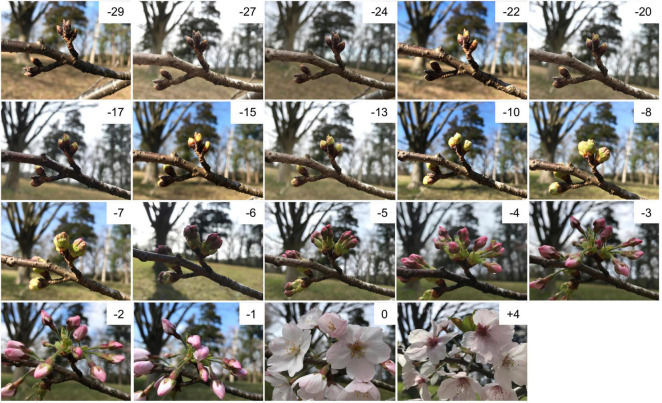
Floral buds and open-flowers of ‘Somei-Yoshino.’ The numbers in each picture indicate days before (–) or after (+) the flowering day (0). Photos were taken at Kazusa DNA Research Institute in 2021.

Seven clonal trees of ‘Somei-Yoshino’ were used in this study. One tree was planted at the Kazusa DNA Research Institute (KDRI; Kisarazu, Chiba, Japan), and three trees each were planted at two different locations: Shimane University (SU; Matsue, Shimane, Japan; trees 1–3) and Kyoto Prefectural University (KPU; Sakyo, Kyoto, Japan; trees A–C). Floral buds and open-flowers were collected in 2019 over an extended time period, ranging from 36 days before flowering (DBF) to 11 days after flowering (DAF) (Figure 1), corresponding to 6 weeks before flowering (WBF) to 2 weeks after flowering (WAF) (Supplementary Table 1). In addition, floral buds of the bud break stage and open-flowers collected in 2020 and 2021 at KDRI, SU, and KPU (Supplementary Table 1) were used for quantitative reverse transcript (RT)-PCR.

### RNA-Seq Analysis

Library preparation and sequencing analysis were performed as described in Shirasawa et al. (2019). In short, total RNA was extracted from the buds and open-flowers with RNeasy Plant Min Kit (Qiagen, Hilden, Germany). The RNA was treated with RNase-free DNase (Promega, Madison, WI, United States) and used for library construction with TruSeq Stranded mRNA Library Prep Kit (Illumina, San Diego, CA, United States). The library was sequenced on NextSeq 500 (Illumina) in paired-end, 76 bp mode.

### RNA Sequence Data Processing

RNA-seq data was analyzed as described previously (Shirasawa et al., 2019). High-quality reads were selected by trimming the adapter sequences using fastx_clipper (parameter, -a AGATCGGAAGAGC) in the FASTX-Toolkit v.0.0.13,^1^ and by deleting low-quality bases using PRINSEQ v.0.20.4 (Schmieder and Edwards, 2011). High-quality reads were mapped to the CYE_r3.1_pseudomolecule sequence (Shirasawa et al., 2019) using HISAT2 v.2.1.0 (Kim et al., 2015), and reads mapped to each gene model were quantified and normalized to determine the number of fragments per kilobase of transcript per million mapped reads (FPKM) using StringTie v.1.3.5 (Pertea et al., 2015) and Ballgown v.2.14.1 (Frazee et al., 2015), as described previously (Pertea et al., 2016). Genes showing a variance of ≥ 1 in expression levels among samples were used for further analysis.

### Gene Ontology Enrichment Analysis of Gene Modules

The weighted gene correlation network analysis (WGCNA; v.1.66) package of R (Langfelder and Horvath, 2008) was used for module detection. The floral bud and open-flower samples were grouped in accordance with gene expression levels using the hierarchical clustering algorithm. Then, highly co-expressed gene clusters were constructed as modules. Genes included in the modules were functionally annotated by performing a BLAST sequence similarity search (Altschul et al., 1997) against the non-redundant (nr) protein database and UniProt database (UniProt Consortium, 2021). GO terms were assigned to genes, and the statistical analysis of the GO term enrichment in each module was performed using Fisher’s exact test implemented in OmicsBox (BioBam, Valencia, Spain). GO maps were drawn with QuickGO (Huntley et al., 2009).

### Quantitative RT-PCR and Establishment a Model for Cherry Blossom Forecasting

Total RNA was extracted from floral buds and open flowers with FavorPrep Plant Total RNA Mini Kit for Woody Plant (Favorgen, Ping-Tung, Taiwan). The PCR mixture (17 μL) contained 250 ng total RNA, 0.8μM primers (Supplementary Table 2), 0.3 μM probes labeled with fluorescent dyes and quenchers at the 5′ and 3′ ends, respectively (Supplementary Table 2), 1 × KAPA Plant PCR buffer (Kapa Biosystems, Wilmington, MA, United States), 1.5 U KAPA 3G Plant polymerase (Kapa Biosystems), and 200 U FastGene Scriptase II (FastGene, Tokyo, Japan). The thermal cycling conditions were as follows: reverse transcription at 42°C for 3 min; initial denaturation at 95°C for 15 s; and 50 cycles of denaturation at 95°C for 5 s, annealing and extension at 60°C for 20 s. The reaction and signal detection were performed on a mobile real-time PCR instrument PicoGene PCR1100 (Nippon Sheet Glass, Tokyo, Japan). Cycle threshold (Ct) values automatically calculated by the PCR instrument were used as the gene expression levels and ΔCt values were calculated as follows:


$$\Delta Ct_{CBFb|CBFg\;}=\;Ct_{CBFb|CBFg\;}-\;Ct_{CBFr}$$

Then, a non-linear regression was used to capture the relationship between the bud stages and the ΔCt values. A statistical model to estimate the ΔCt values on the flowering day was established by a generalized linear regression to predict the flowering date. The calculation was performed by R (R Core Team, 2021).

## Results

### RNA-Seq Analysis and Quantification of Gene Expression Levels

A total of 94 samples were collected from flowering cherry trees at KDRI, SU, and KPU over a period of 47 days (i.e., 36 days before anthesis to 11 days post-anthesis) in 2019 (Figure 1 and Supplementary Table 1). Total RNA was extracted from the samples and subjected to RNA-seq analysis, which returned approximately 4.7 million reads per sample. Nucleotide sequence data of the RNA-seq were deposited in the DDBJ Sequence Read Archive (accession numbers DRA012801, DRA012802, and DRA012803). We also utilized the RNA-seq data generated previously (Shirasawa et al., 2019; DRA accession number DRA008100) from samples collected at KDRI in 2017. The RNA-seq reads from both datasets were mapped on to the genome sequence of ‘Somei-Yoshino’ (Shirasawa et al., 2019), and expression levels were normalized in all samples based on the total reads to obtain FPKM values for each gene across the samples. Of the 95,076 genes predicted in the ‘Somei-Yoshino’ genome (Shirasawa et al., 2019), a total of 29,712 genes (31.3%) were expressed across the different samples, with a variance of ≥ 1.

### Gene Module Detection

**FIGURE 2:**
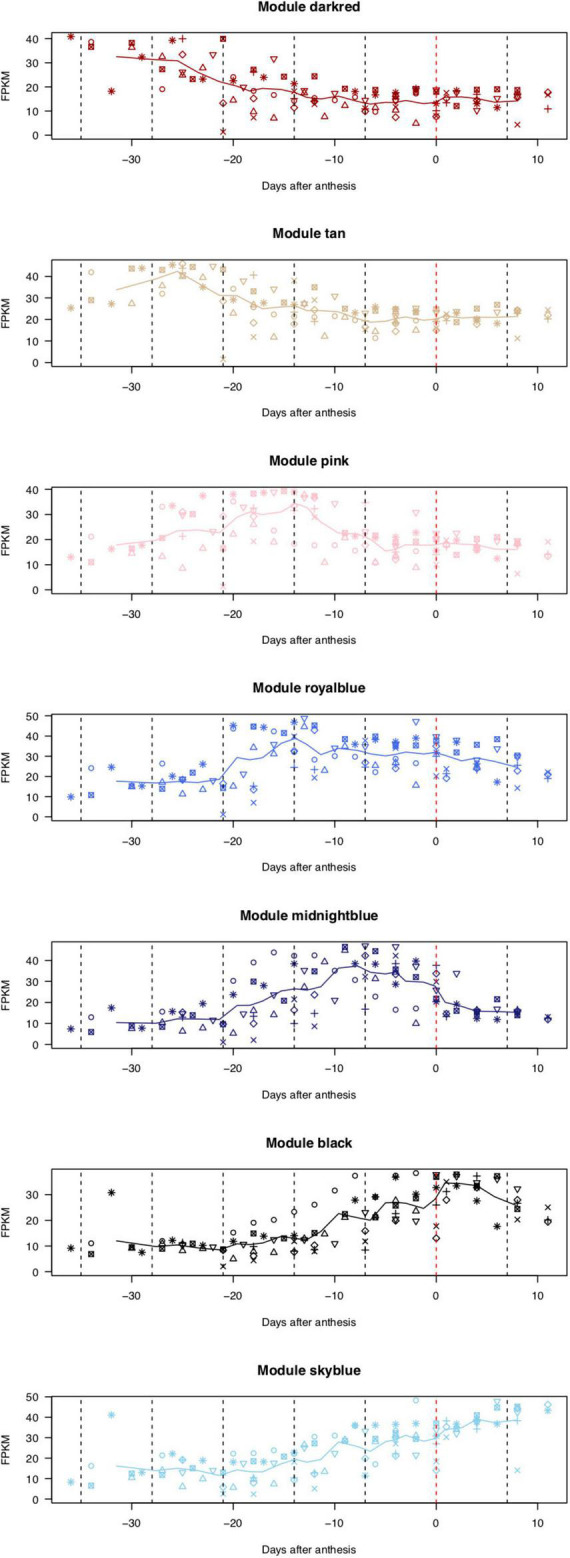
Expression patterns of genes categorized into seven modules. X-axis indicates the number of days before (–) or after (+) the flowering day (0). Lines show moving averages of gene expression (sliding window size = 8, walking speed = 4). Symbols indicate biological replicates: KDRI in 2017 (circle); KDRI in 2019 (triangle); SU1 in 2019 (plus); SU2 in 2019 (cross); SU3 in 2019 (diamond); KPUA in 2019 (upside-down triangle); KPUB in 2019 (square cross); and KPUC in 2019 (star).

On the basis of the expression patterns of 29,712 genes, one floral bud sample collected at 27 days before anthesis from the KDRI location was identified as an outlier (KDRI 2019-03-04 sample) and therefore was excluded from further analyses (Supplementary Figure 1). The remaining 93 samples were roughly clustered into 53 highly co-expressed gene modules, based on the expression patterns of 29,712 genes (Supplementary Figure 2), which were further classed into four groups, based on eigengenes (Supplementary Figure 3). Genes in 7 out of 53 modules were prominently expressed at specific days before or after anthesis (Figure 2): dark-red module [110 genes expressed at 4–5 weeks before flowering (WBF)], tan module (267 genes, 4 WBF), pink module (332 genes, 3 WBF), royal blue module (127 genes, 2–3 WBF), midnight blue module (176 genes, 1–2 WBF), black module (457 genes, at flowering days), and sky-blue module [72 genes, 1–2 weeks after flowering (WAF)]. Of the remaining 46 modules, 24 module genes exhibited constitutive expression across the samples and 22 modules showed different patterns across trees, places, and/or years (Supplementary Figure 2).

### Gene Ontology Enrichment Analysis

To identify the GO terms enriched in each module, the ratios of GO terms in each gene module were compared with those of the remaining gene set (Table 1, Supplementary Table 3 and Supplementary Figure 4). At 4-5 WBF, eight GO terms related to development, e.g., five biological processes (BP), one molecular function (MF), and two cellular components (CC), were enriched. At the following stages, 17 (morphogenesis), 17 (metabolism), 15 (cell wall organization), and 4 (gametophyte development) GO terms were overrepresented at 4 WBF, 3 WBF, 2–3 WBF, and 1–2 WBF, respectively. At the flowering stage, 10 GO terms related to enzyme regulators were enriched. At the 1–2 WAF, overrepresented GO terms were expanded to 24 biological regulations including responses to stimuli, signal transductions, protein modifications, and metabolisms.

**TABLE 1:** Gene Ontology (GO) terms enriched at the different flowering stages.

Module	No. of genes	Stage^a^	Biological process (BP)	Molecular function (MF)	Cellular component (CC)
Dark-red	110	4–5 WBF	“Developmental process” related GO terms including “aging,” “embryo development,” “developmental maturation,” “developmental process,” and “anatomical structure development”	“Oxidoreductase activity”	“Plasma membrane” and “cell periphery”
Tan	267	4 WBF	“Anatomical structure formation involved in morphogenesis,” “anatomical structure development,” “sulfur compound metabolic process,” “biosynthetic process,” “developmental process,” and “cell wall organization or biogenesis”	“DNA binding,” “DNA-binding transcription factor activity,” “catalytic activity,” “oxidoreductase activity,” “transferase activity, transferring acyl groups,” “hydrolase activity, acting on glycosyl bonds,” “lyase activity,” and “transcription regulator activity”	“Extracellular region”
Pink	332	3 WBF	“Lipid metabolic process,” “autophagy,” “small molecule metabolic process,, and “process utilizing autophagic mechanism”	“Catalytic activity,” “transporter activity,” “lipid binding,” “oxidoreductase activity,” “transferase activity, transferring acyl groups,” and “transmembrane transporter activity”activity, transmembrane transporter activity	“Extracellular region,” “extracellular space,” “vacuole,” “plasma membrane,” “membrane,” and “cell periphery”
Royal blue	127	2–3 WBF	“Mitotic cell cycle,, “carbohydrate metabolic process,” “catabolic process,” “secondary metabolic process,” “cell division,” and “cell wall organization or biogenesis”	Not detected	“Extracellular region,” “cell wall,” “Golgi apparatus,” “plasma membrane,” “Endomembrane system,” “membrane,” “external encapsulating structure,” “cell periphery,” and “cellular anatomical entity”
Midnight blue	176	1–2 WBF	“Pollen development” and “gametophyte development”	“Transporter activity” and “transmembrane transporter activity”	Not detected
Black	457	Flowering day	“Carbohydrate metabolic process,” “cytoskeleton organization,” “catabolic process,” and “cell wall organization or biogenesis”	“Lipid binding,” “lyase activity,” “enzyme regulator activity,” and “molecular function regulator”	“Extracellular region” and “extracellular space”
Sky-blue	72	1–2 WAF	“Carbohydrate metabolic process,” “cellular protein modification process,” “response to stress,” “cell communication,” “signal transduction,” “metabolic process,” “catabolic process,” “signaling,” “protein modification process,” “macromolecule modification,” “primary metabolic process,” “regulation of biological process,” “regulation of cellular process,” “response to stimulus,” “cellular response to stimulus,” “biological regulation,” “cell wall organization or biogenesis,” and “organic substance metabolic process”	“Lipid binding,” “hydrolase activity, acting on glycosyl bonds,” “enzyme regulator activity,” and “molecular function regulator”	“Nucleus”

*^a^WBF, weeks before flowering; WAF, weeks after flowering.*

### Genetic Mechanisms Underlying Blooming in Flowering Cherry

**FIGURE 3:**
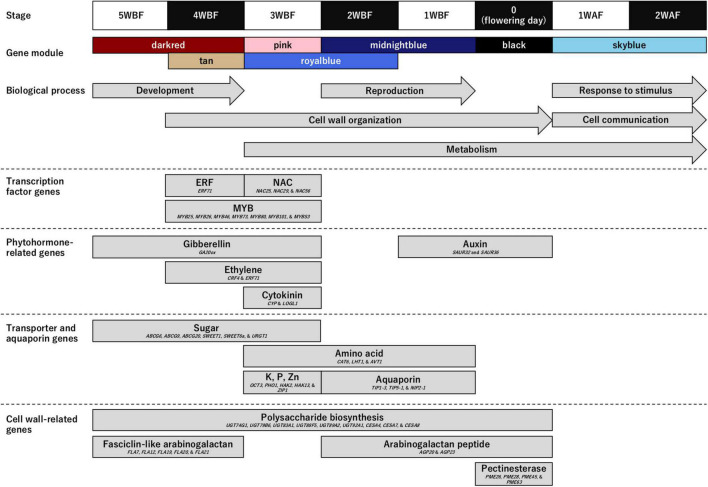
Genes enriched in the biological process (BP) category and involved in the flower opening mechanism in ‘Somei-Yoshino.’ Upper panels show the time frame [from 5 weeks before flowering (WBF) to 2 weeks after flowering (WAF)] and the corresponding gene modules. Arrows indicate the Gene Ontology (GO) terms enriched in the BP category (see Table 1). Boxes indicate the properties of the genes highly expressed in the seven characterized modules (see Supplementary Table 3).

Owing to several recent knowledge on the flower opening (van Doorn and Kamdee, 2014; Chen et al., 2020; Zhao et al., 2020; Cheng et al., 2021; Li et al., 2021; Sun et al., 2021), we hypothesized that transcription factors would be activated by phytohormone induced by environmental stimuli and regulate transporters and aquaporins to expand floral organ cells associating cell wall organization. Among them, we assumed that cell wall organization might be a direct mechanical driver for petal movement to open flowers. Therefore, to gain insights into the genetic mechanisms regulating blooming in flowering cherry, we focused on genes categorized in four functional categories (Figure 3 and Supplementary Tables 3, 4): (1) transcription factor genes; (2) phytohormone-related genes; (3) transporter and aquaporin genes; and (4) cell wall-related genes.

#### Transcription Factor Genes

Genes encoding three types of transcription factors that trigger blooming were predominant during the flowering period. The *MYB* transcription factor genes (*MYB25*, *MYB26*, *MYB46*, *MYB73*, *MYB80*, *MYB101*, and *MYBS3*) were overrepresented from 4 to 3 WBF, while the ethylene-responsive transcription factor (*ERF71*) genes and *NAC* transcription factor genes (*NAC25*, *NAC29*, and *NAC56*) were expressed at 4 and 3 WBF, respectively.

#### Phytohormone-Related Genes

Genes involved in biosynthesis and signal transduction pathways of gibberellin (*GA20ox*), ethylene (*CRF4* and *ERF71*), and cytokinin (*CYP* and *LOGL1*) were enriched at 3–5 WBF, 3–4 WBF, and 3 WBF, respectively. Auxin-related genes (*SAUR32* and *SAUR36*) were, on the other hand, expressed at 1 WBF and at the flowering days.

#### Transporter and Aquaporin Genes

Genes encoding sugar and inorganic transporters and aquaporins that affect the turgor and osmotic pressure of cells and vacuoles were constitutively expressed from 5 WBF to the day for flowering. Among these, genes encoding 10 types of transporters [ABC transporter G family members (*ABCG6*, *ABCG9*, and *ABCG20*), bidirectional sugar transporters (*SWEET1* and *SWEET6a*), cationic amino acid transporters (*CAT6*), lysine histidine transporters (*LHT1*), organic cation/carnitine transporters (*OCT3*), phosphate transporters (*PHO1*), potassium transporters (*HAK2* and *HAK13*), UDP-galactose transporters (*URGT1*), vacuolar amino acid transporters (*AVT1*), and zinc transporters (*ZIP1*)] were overrepresented at 3 WBF. Aquaporin genes (*TIP1-3*, *TIP5-1*, and *NIP2-1*) were overrepresented at 1–2 WAF.

#### Cell Wall-Related Genes

Cell wall organization followed by cell expansions actuate petal movement, leading to flower opening. Genes encoding polysaccharide biosynthesis enzymes including UDP-glycosyltransferases (*UGT74G1*, *UGT79B6*, *UGT83A1*, *UGT88F5*, *UGT89A2*, and *UGT92A1*) and cellulose synthases (*CESA4*, *CESA7*, and *CESA8*) were expressed from 5 WBF to the day for flowering. Genes encoding fasciclin-like arabinogalactan proteins (*FLA7*, *FLA12*, *FLA19*, *FLA20*, and *FLA21*) were mainly expressed at the initial stage of the flowering period, while those encoding arabinogalactan peptides (*AGP20* and *AGP23*) were expressed at the flowering days. Pectinesterase genes (*PME26*, *PME28*, *PME45*, and *PME63*) were overrepresented at the flowering days.

### Cherry Blossom Forecast Model Based on Transcriptome

**FIGURE 4:**
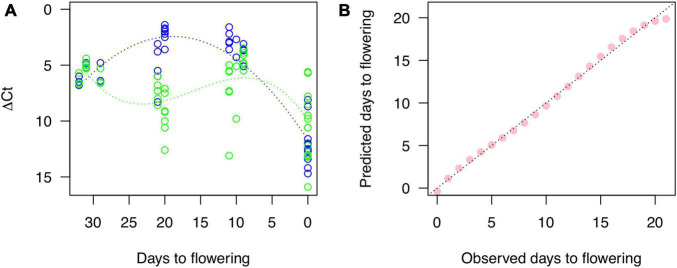
Gene expression quantified by RT-PCR and a fitness of the model for cherry blossom forecast based on the RNA expression. **(A)** Blue and green dots show expression level of genes for CYE_r3.1SPA4_g005220.1 (CBFb) and CYE_r3.1SPA0_g059890.1 (CBFg), respectively, related to a control gene, CYE_r3.1SPA3_g018730.1 (CBFr). Blue and green lines indicate non-linear regression curves. **(B)** Correlation between observed days to flowering and those predicted by the model for cherry blossom forecast established with a generalized linear regression.

Two high-expressed genes were selected as *Cherry Blossom Forecast (CBF)* genes from the modules of pink (CYE_r3.1SPA4_g005220.1 as *CBFb*) and midnight blue (CYE_r3.1SPA0_g059890.1 as *CBFg*), which were similar with glycine-rich cell wall structural protein 2-like (XP_021825805) and subtilisin-like protease SBT4.3 (XP_021832757), respectively. In addition, as a positive control, one gene (CYE_r3.1SPA3_g018730.1 as *CBFr*) for ribulose bisphosphate carboxylase small chain (PQQ09003) was selected from the turquoise module, which expression was constantly increased along the stages (Supplementary Figure 2). The Ct values representing RNA expression levels of the three genes were quantified and ΔCt values for each target were calculated. A non-linear regression gave quadratic and cubic function curves to the ΔCt of *CBFb* and *CBFg*, respectively (Figure 4A). The peaks of the curves were expectedly at 20 DBF (*CBFb*) and 10 DBF (*CBFg*). Then, we used a generalized linear regression to establish a statistical model to predict days to flowering as the following formula:


$$D\,a\,y\,s\,t\,o\,f\,l\,o\,w\,e\,r\,i\,n\,g∼3.56\cdot △\,C\,t_{C\,B\,F\,g}-0.59\cdot △\,C\,t_{C\,B\,F\,b}-0.22\cdot △\,C\,t_{C\,B\,F\,g}\cdot △\,C\,t_{C\,B\,F\,b}-3.70$$

The predicted days well fit to observed days ranging from 21 DBF to the flowering day (Figure 4B).

## Discussion

Time-course RNA-seq analysis revealed the dynamics of the transcriptome of floral buds and flowers of the flowering cherry cultivar ‘Somei-Yoshino.’ Subsequent WGCNA of the RNA-seq data indicated that 1,541 genes belonging to seven modules were involved in blooming at stages from 4 to 5 WBF to 1–2 WAF (Figure 2 and Supplementary Tables 3, 4). In addition, 24 and 22 modules were also found to possess constitutive expressed genes and differential expressed genes across trees, places, and/or years (Supplementary Figure 2), respectively. The latter differential expressed genes might be regulated by environment conditions, e.g., photoperiod and abiotic stresses (Lloret et al., 2017; Tylewicz et al., 2018). These genes could be useful to understand phenotypic variations controlled by gene–environment interaction.

In accordance with the functional annotations of the genes in the seven modules, those involved in floral bud and open-flower development as well as blooming were identified in flowering cherry (Figure 3). The genes as well as GO terms enriched in the flowering stages well explained the flower development physiology in Rosaceae. Floral bud development potentially resumes from 4 to 5 WBF, with the *GA20ox* for gibberellin biosynthesis controlling sugar and polysaccharide metabolism to initiate the development of floral organs in the post-dormancy stage. Subsequently, at 3–4 WBF, cell wall organization and metabolism might be initiated. Ethylene (*CRF4* and *ERF71*) and cytokinin (*CYP* and *LOGL1*) likely accelerate the expression of *MYB26* and *MYB46* transcription factor genes, resulting in the initiation of cell wall organization and cell division (Nakano et al., 2015). *MYB25*, *MYB80*, *MYB101*, *NAC25*, *NAC29*, and *NAC56* genes are also involved in gametophyte or ovule integument development (Kunieda et al., 2008; Renak et al., 2012). According to the observations in sweet cherry (*Prunus avium*), the 3–4 WBF timepoint corresponds to the formation of tetrads or microspores after meiosis in the pollen (Fadon et al., 2019). At this stage, *CAT6*, *LHT1*, and *AVT1* genes for amino acid metabolism is also upregulated. Metabolome analysis of the flower buds of Japanese apricot (*Prunus mume*) revealed significant changes in the abundance of several amino acids before the bud break stage (Zhuang et al., 2015). Amino acids play important roles in bud break. At 1–2 WBF, the reproduction process, together with the cell wall organization and metabolism, has been likely activated. Auxin-related genes *SAUR32* and *SAUR36* were also activated at this stage in this current study. On the flowering day, we speculate that the auxin signal alters pectinesterase gene expression such as *PME26*, *PME28*, *PME45*, and *PME63*, all of which act in the modification of cell walls via demethylesterification of cell wall pectin, to initiate cell wall remodeling, leading to rapid petal enlargement and movement, which is the most dynamic movement in flowering plants (Habu and Tao, 2014; Nakano et al., 2015). The high-level expression of aquaporin genes, *TIP1-3*, *TIP5-1*, and *NIP2-1*, which encode water channel proteins localized to the tonoplast membranes, on the day of flowering might facilitate the water flux into the petals to allow smooth petal movement (Azad et al., 2013). The relationship between auxin signaling and expression of pectinesterase genes, e.g., *PME26*, *PME28*, *PME45*, and *PME63*, has been demonstrated in the ripening process of strawberry fruit (Castillejo et al., 2004). Similar hormonal regulations might be conserved in the Rosaceae species, even though the different organs. The role of the auxin signal in the dynamic movement of petals during flower opening needs further investigation. After blooming, genes involved in the stimulus response and cell-to-cell communication are expressed in the flower, especially in the pistil and ovary, which might reflect the pollination and fertilization process, even though ‘Somei-Yoshino’ is self-incompatible. Similar transcriptome profiles were observed in other *Prunus* species (Habu and Tao, 2014; Gómez et al., 2019; Iqbal et al., 2021). Compared with the molecular mechanism on flower opening process in this study (Figure 3), similar processes have been activated and modulated even in dormancy period, with genes for sugar synthesis and mobilization, lipid peroxidation, coumarate metabolism, transmembrane transport, cell wall remodeling, and ABA synthesis and signaling (Prudencio et al., 2021) as well as gametophyte development in *Prunus* species (Julian et al., 2011; Rios et al., 2013). One exceptional difference was that genes for “cell wall organization or biogenesis” (GO:0071554) were activated in the flower opening process, which would be required for the rapid cell expansion in the exponential enlargement of floral organs and petal emergence movement like fruit enlargement (Zanchin et al., 1994).

Because the flowering time of ‘Somei-Yoshino’ is important for the tourism industry in Japan, methods for predicting the flowering date of this cultivar have been developed in accordance with the cumulative temperature before flower opening (Aono and Murakami, 2017). Gene expression analysis could upgrade the current forecasting model, for which genes expressed on specific days before flowering could be employed as diagnostic markers. For this purpose, genes expressed stably across different conditions (trees, places, and years) are required rather than those responsive to environments. In this study, stable genes were selected from RNA-Seq data in multiple environmental conditions (seven trees at Chiba, Kyoto, and Shimane in 2017 and 2019) (Figure 2) and the expression was confirmed with RT-PCR using different samples collected in 2020 and 2021 (Figure 4A). Based on the robust genes, a statistical model for cherry bloom forecast was established. Besides, since diagnostic markers should be detected easily within a short time, easy-to-use target-RNA qualifying methods are required. Practical RNA quantification methods, such as real-time quantitative PCR and the next-generation sequencing (NGS)-based RNA-seq, are not suitable due to time-consuming and labor-intensive. In this study, RNA expression was quantified with a mobile PCR instrument, which can be used at any time and place. Another issue to be solved would be RNA extraction from plant samples including flowering buds without any laboratory equipment.

Comparative time-course transcriptome analysis and statistical models based on the transcriptome would enable the selection of high-confidence diagnostic markers for various purposes. For example, time-course transcriptome analysis can be applied to floral buds of vegetable and fruit crops and cut flowers. Moreover, this comparative analysis is not only limited to the selection of markers for forecasting flowering time but is also applicable to the prediction of disease, fertilization, and harvest time in the field. Indeed, transcriptome profiling has been employed to monitor nutritional responses and adaptation in rice (Takehisa and Sato, 2021).

Overall, this study provides insights into the genetic mechanisms controlling petal movement and blooming in cherry. Further studies are required to connect the genetic insights with the physiological mechanisms (van Doorn and Van Meeteren, 2003). Once the connection or relationship is validated, the transcriptome-based prediction would serve as a powerful tool for monitoring the plant phenotype under controlled cultivation conditions as well as in the field (Figure 4B). For example, genome-based prediction has been used to predict progeny phenotypes in breeding programs. The transcriptome- and genome-based predictions would promise high-confidence forecasting plant traits in the near and distant future, respectively.

## Data Availability Statement

The datasets presented in this study can be found in online repositories. The names of the repository/repositories and accession number(s) can be found in the article/Supplementary Material.

## Author Contributions

KS, TE, and AI conceived this study, collected the samples, analyzed, and interpreted the data. KS and SI performed the experiments and collected the data. KS wrote the manuscript with contributions from TE. All authors read and approved the final manuscript.

## Conflict of Interest

The authors declare that the research was conducted in the absence of any commercial or financial relationships that could be construed as a potential conflict of interest.

## Publisher’s Note

All claims expressed in this article are solely those of the authors and do not necessarily represent those of their affiliated organizations, or those of the publisher, the editors and the reviewers. Any product that may be evaluated in this article, or claim that may be made by its manufacturer, is not guaranteed or endorsed by the publisher.

## Footnotes

1
http://hannonlab.cshl.edu/fastx_toolkit
