# Supplementary material for: Cherry Blossom Forecast Based on Transcriptome of Floral Organs Approaching Blooming in the Flowering Cherry (Cerasus × yedoensis) Cultivar ‘Somei-Yoshino’
Source: Front Plant Sci. 2022 Jan 26;13:802203. doi: 10.3389/fpls.2022.802203 (PMC8825344; doi:10.3389/fpls.2022.802203)
Supplement: Supplementary file 1 [file Data_Sheet_1.PDF]

Supplementary Information

Cherry Blossom Forecast Based on Transcriptome Dynamics of Floral Organs Approaching Blooming in the Flowering Cherry (*Cerasus* × *yedoensis*) Cultivar 'Somei-Yoshino'

Kenta Shirasawa[1*], Tomoya Esumi[2], Akihiro Itai[3], Sachiko Isobe[1]

[1]Laboratory of Plant Genetics and Genomics, Department of Frontier Research and Development, Kazusa DNA Research Institute, Kisarazu, Chiba 292-0818, Japan

[2]Laboratory of Pomology & Viticulture, Department of Agricultural and Life Sciences, Shimane University, Matsue, Shimane 690-8504, Japan

[3]Laboratory of Plant Resource Science, Department of Agricultural and Life Science, Kyoto Prefectural University, Sakyo, Kyoto 606-8522, Japan

*** Correspondence:**
Kenta Shirasawa
shirasaw@kazusa.or.jp

Supplementary Table S1 Sampling dates of 'Somei-Yoshino' floral buds and flowers.

Supplementary Table S2 Gene modules overrepresented at different flowering stages.

Supplementary Table S3 Genes and annotations overrepresented in the seven modules.

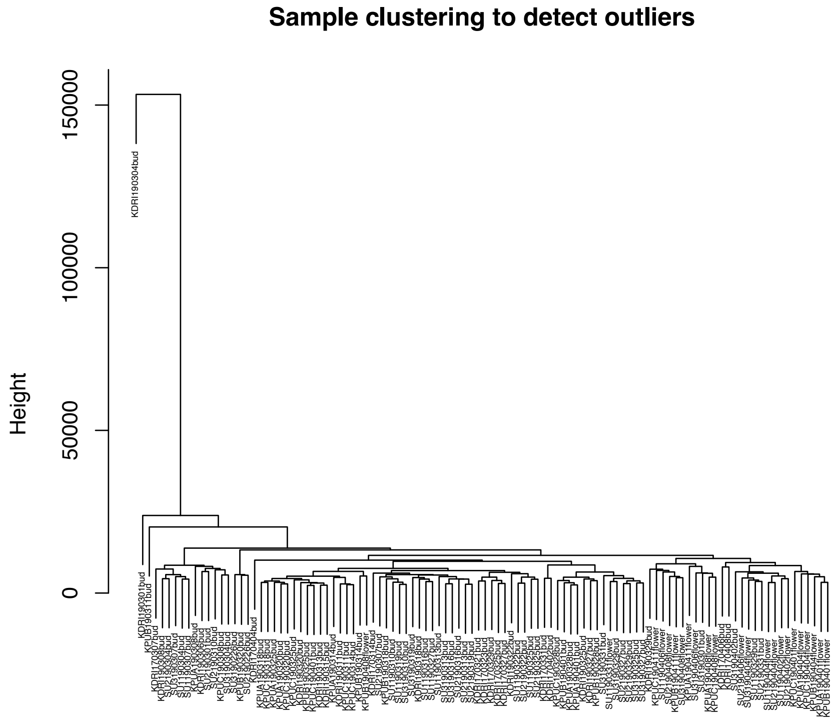

Supplementary Figure S1 Sample clustering used to identify outliers.

A

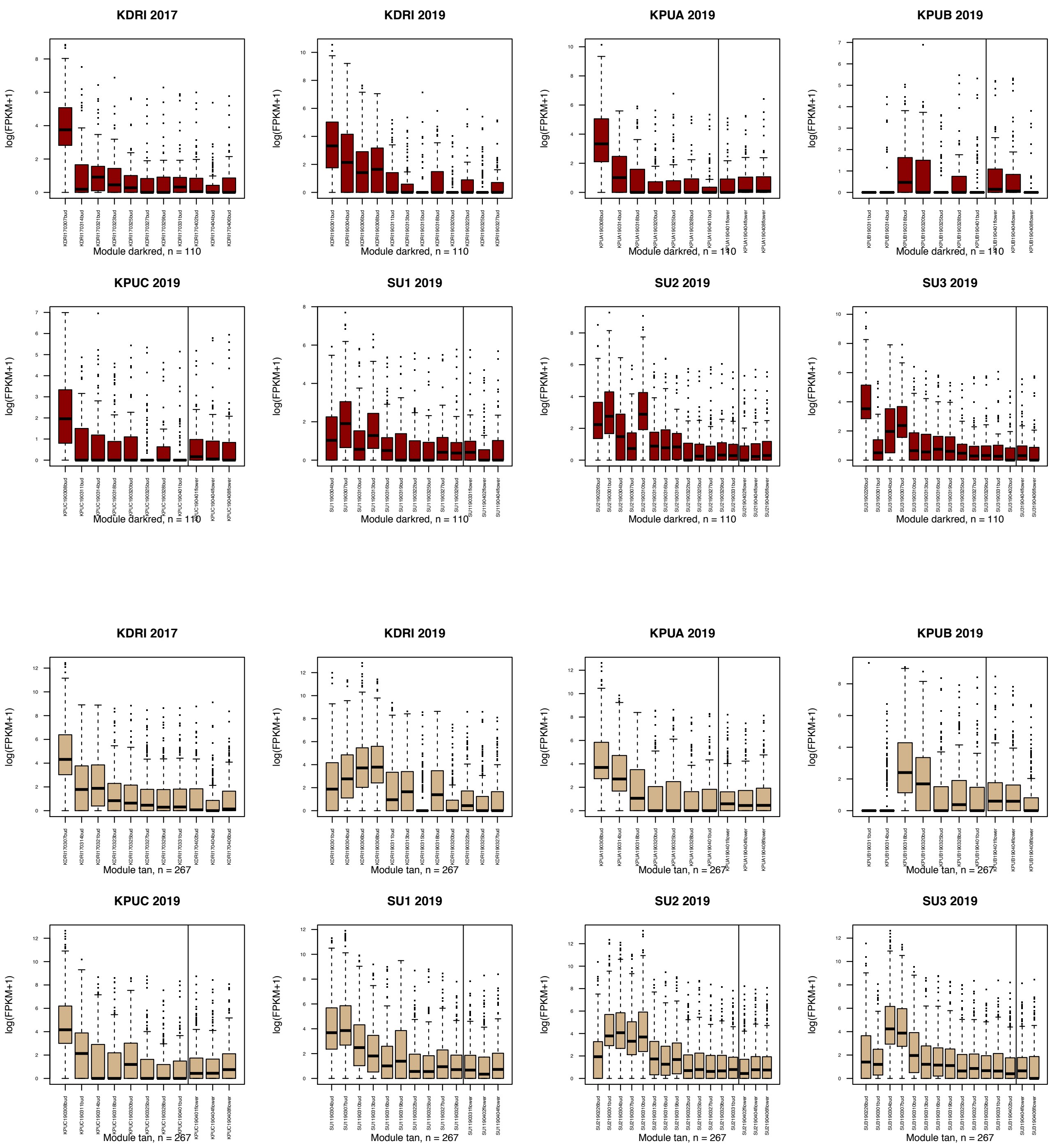

Supplementary Figure S2 Expression patterns of gene modules, based on weighted gene correlation network analysis (WGCNA). Vertical lines indicate the flowering day. A. Gene modules expressed at specific days before or after anthesis, B. Gene modules expressed constitutively, C. Gene modules expressed in different patterns in trees, places, and/or years, and D. Genes not clustered in any modules.

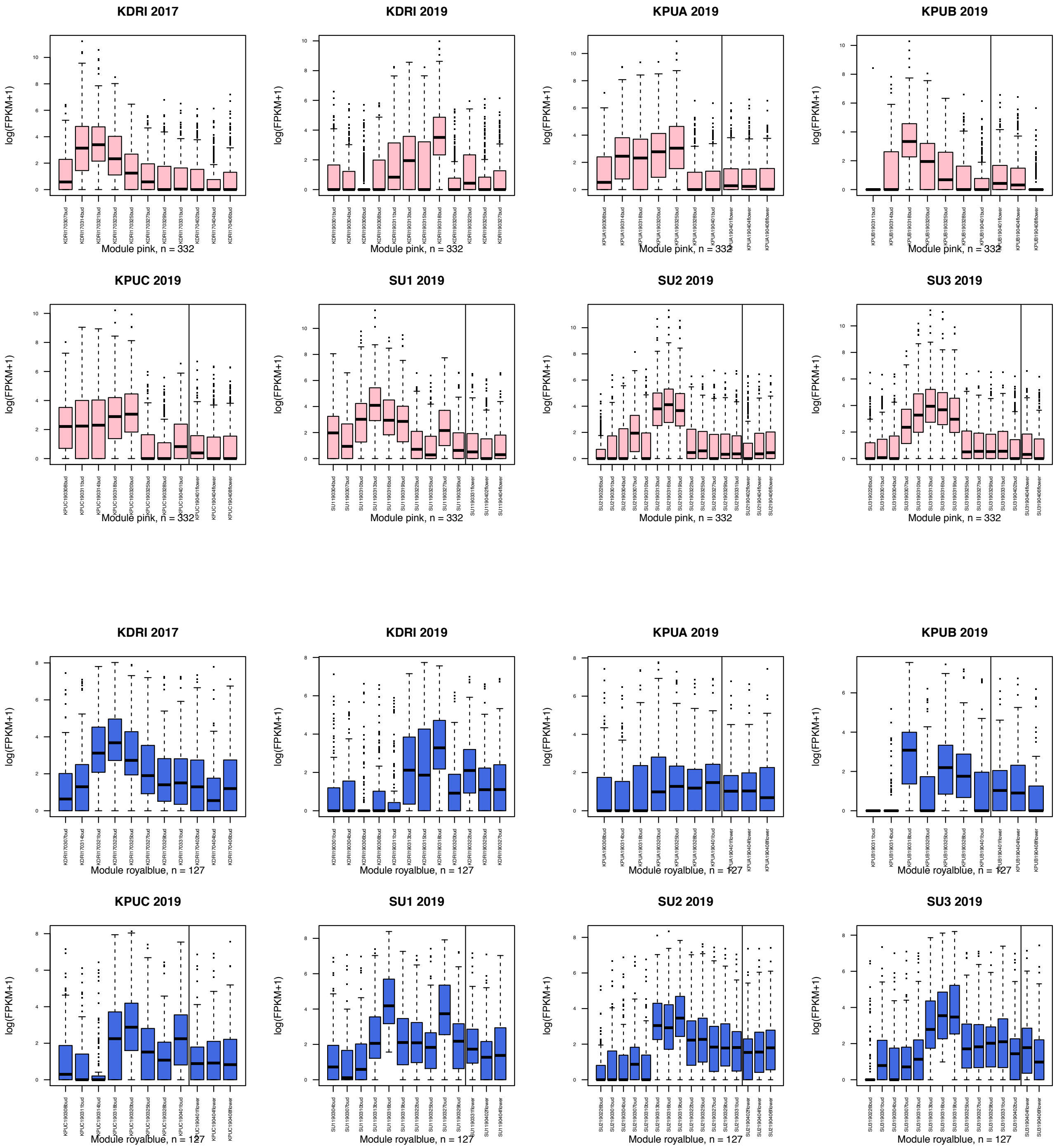

Supplementary Figure S2 (Continued.)

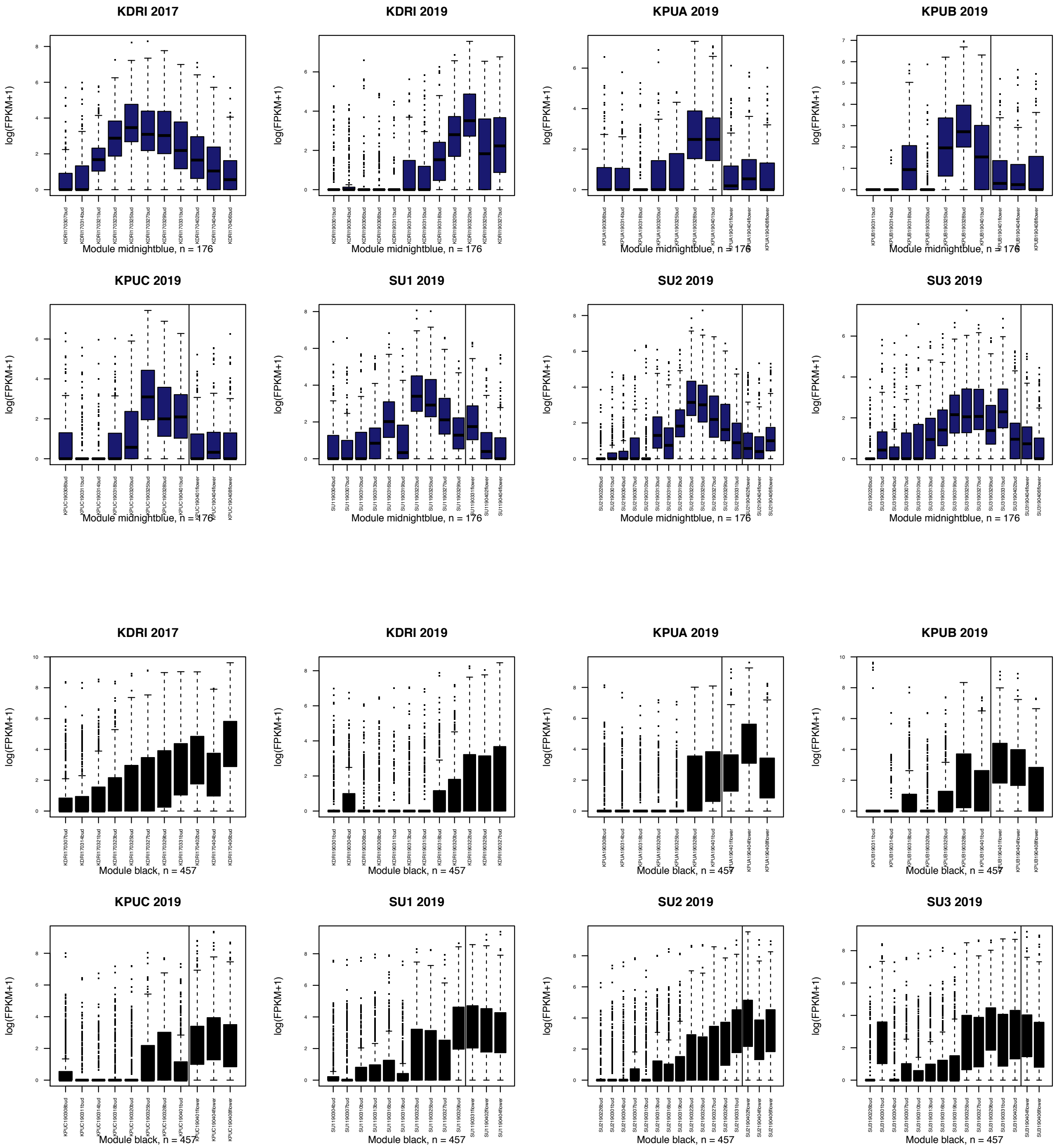

Supplementary Figure S2 (Continued.)

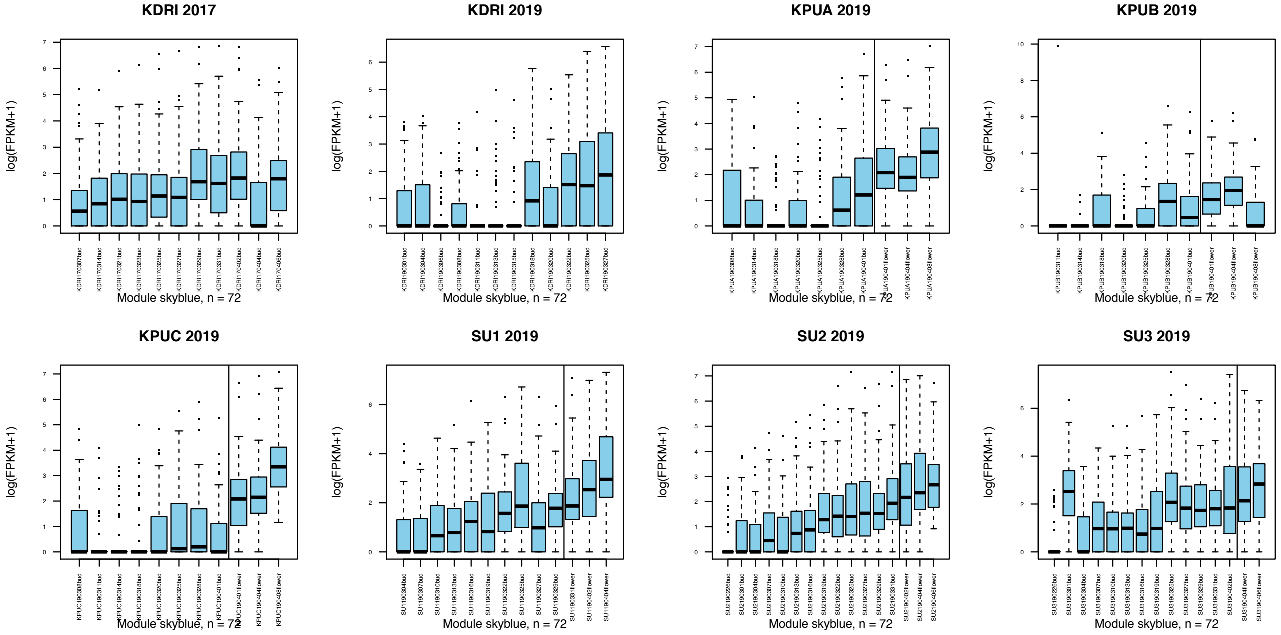

B

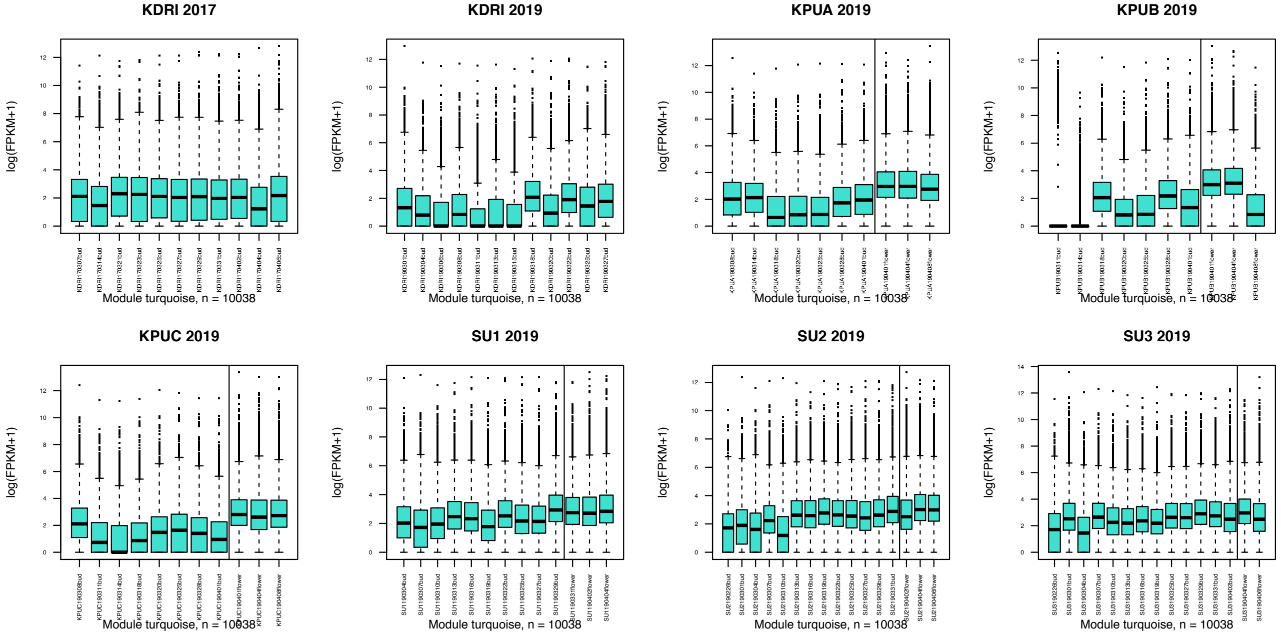

Supplementary Figure S2 (Continued.)

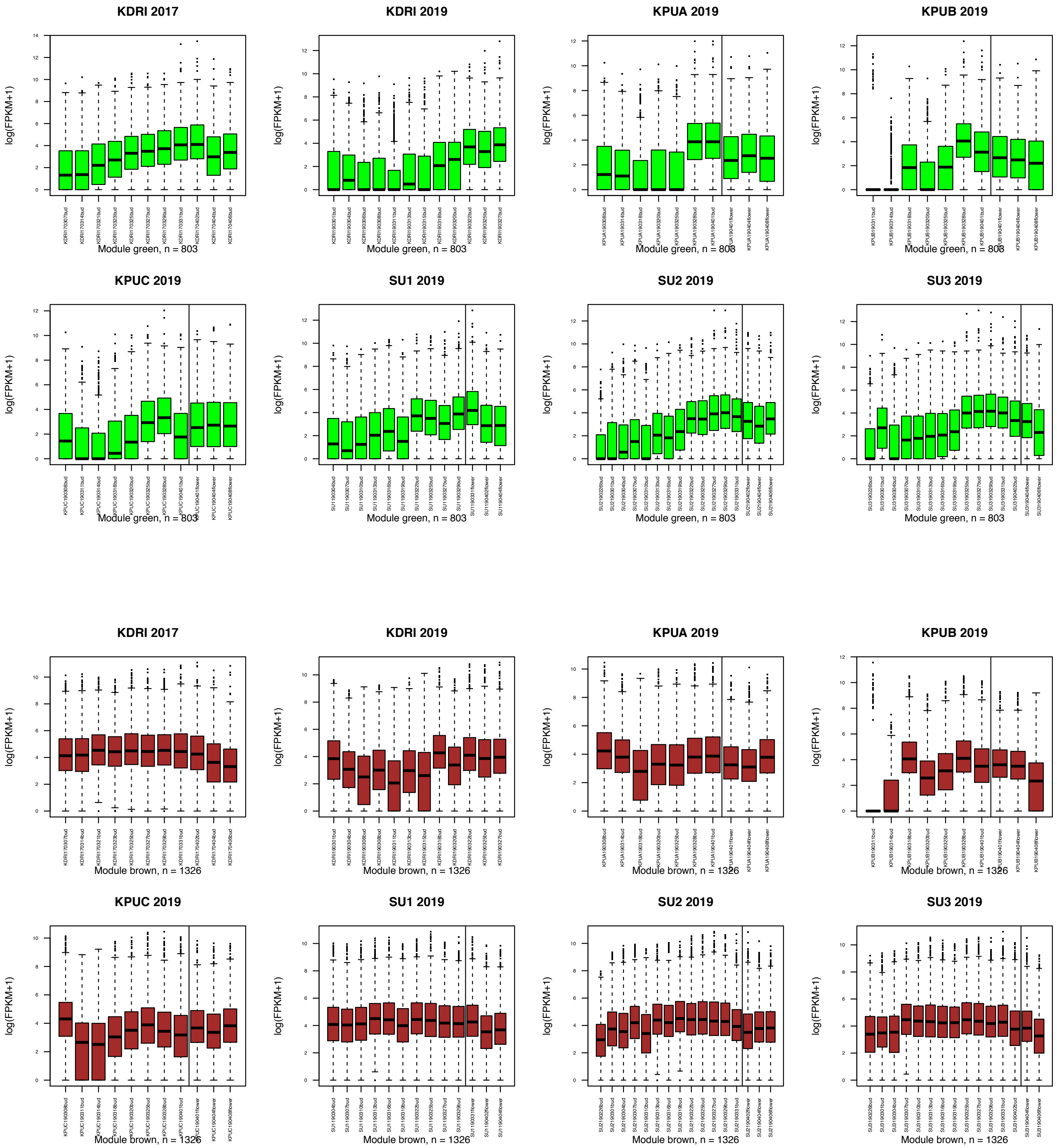

Supplementary Figure S2 (Continued.)

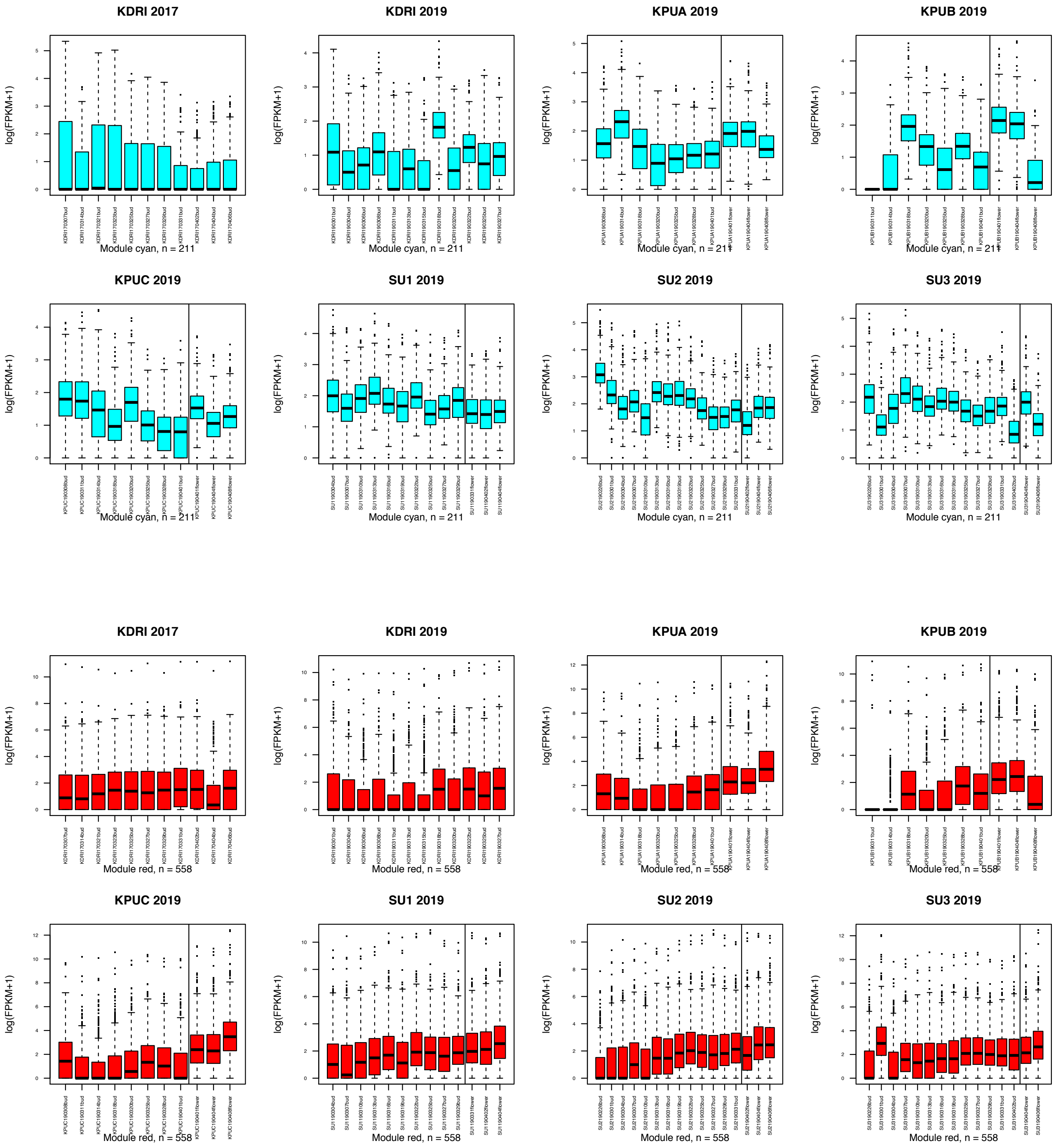

Supplementary Figure S2 (Continued.)

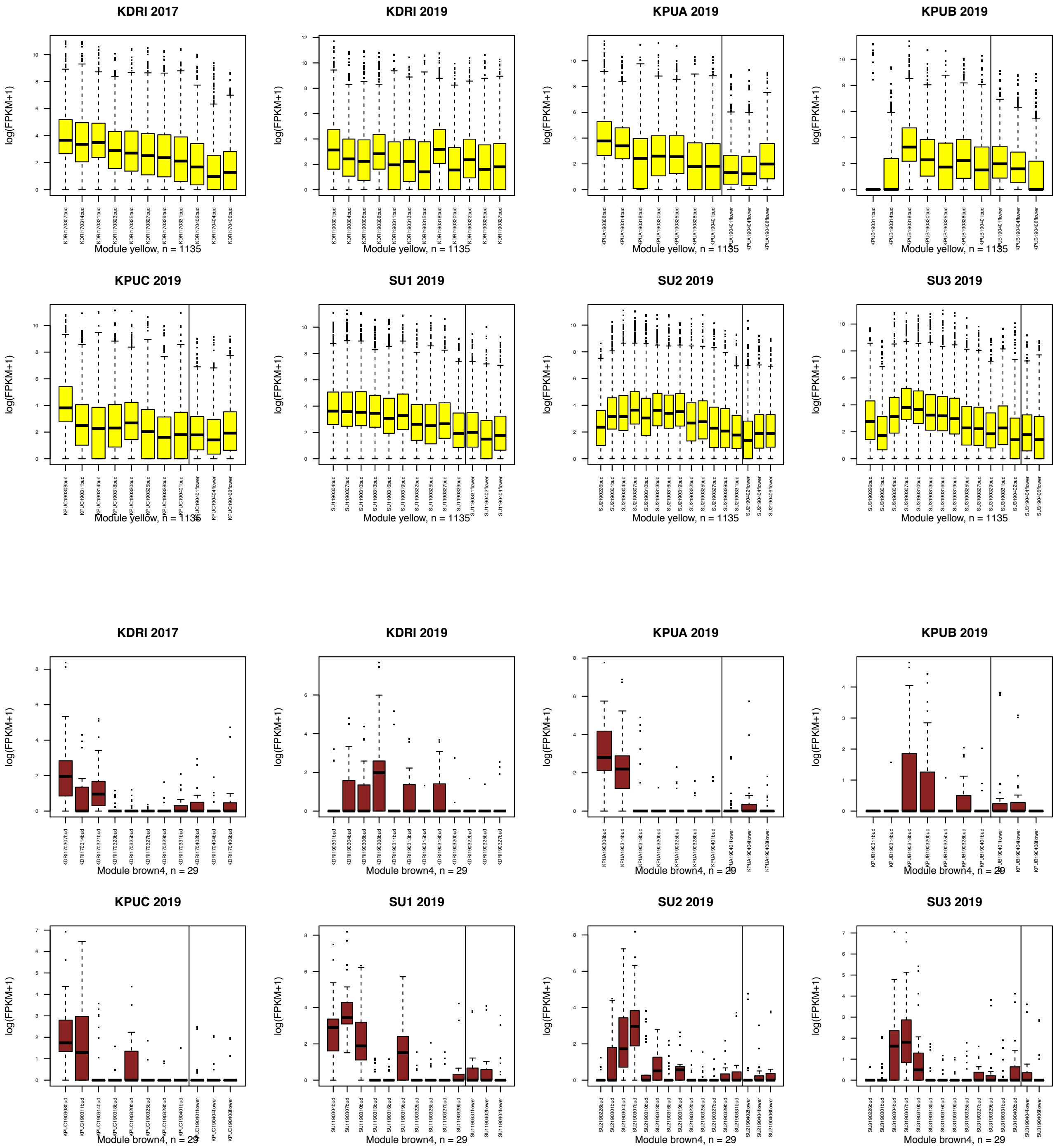

Supplementary Figure S2 (Continued.)

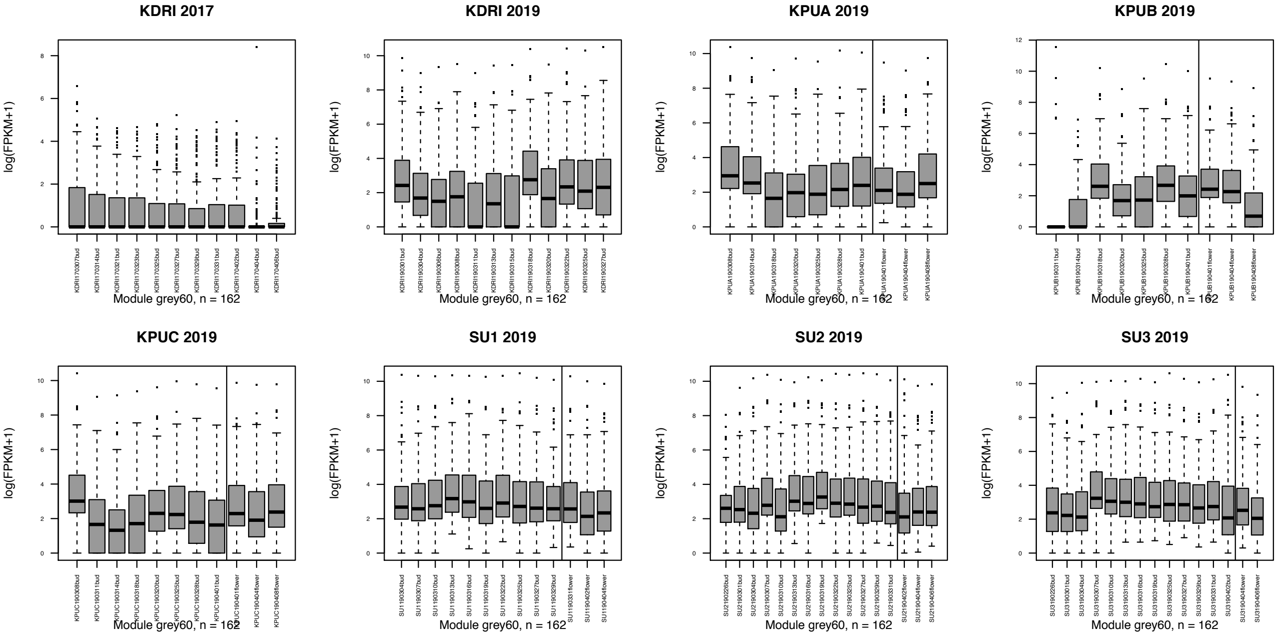

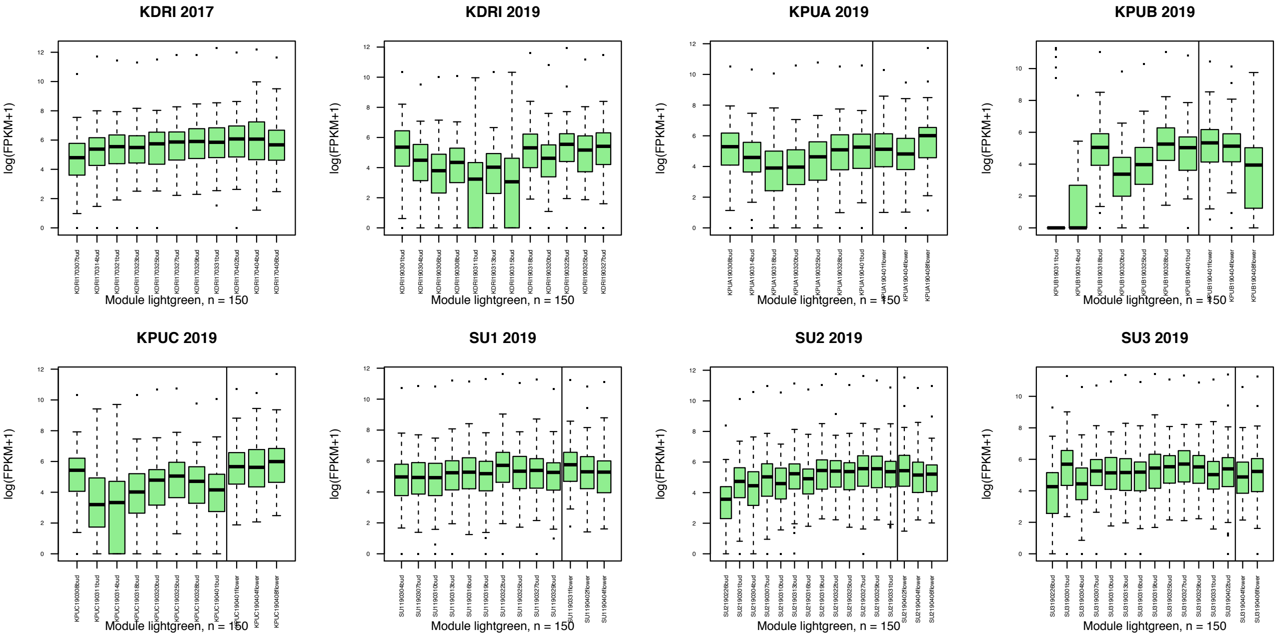

Supplementary Figure S2 (Continued.)

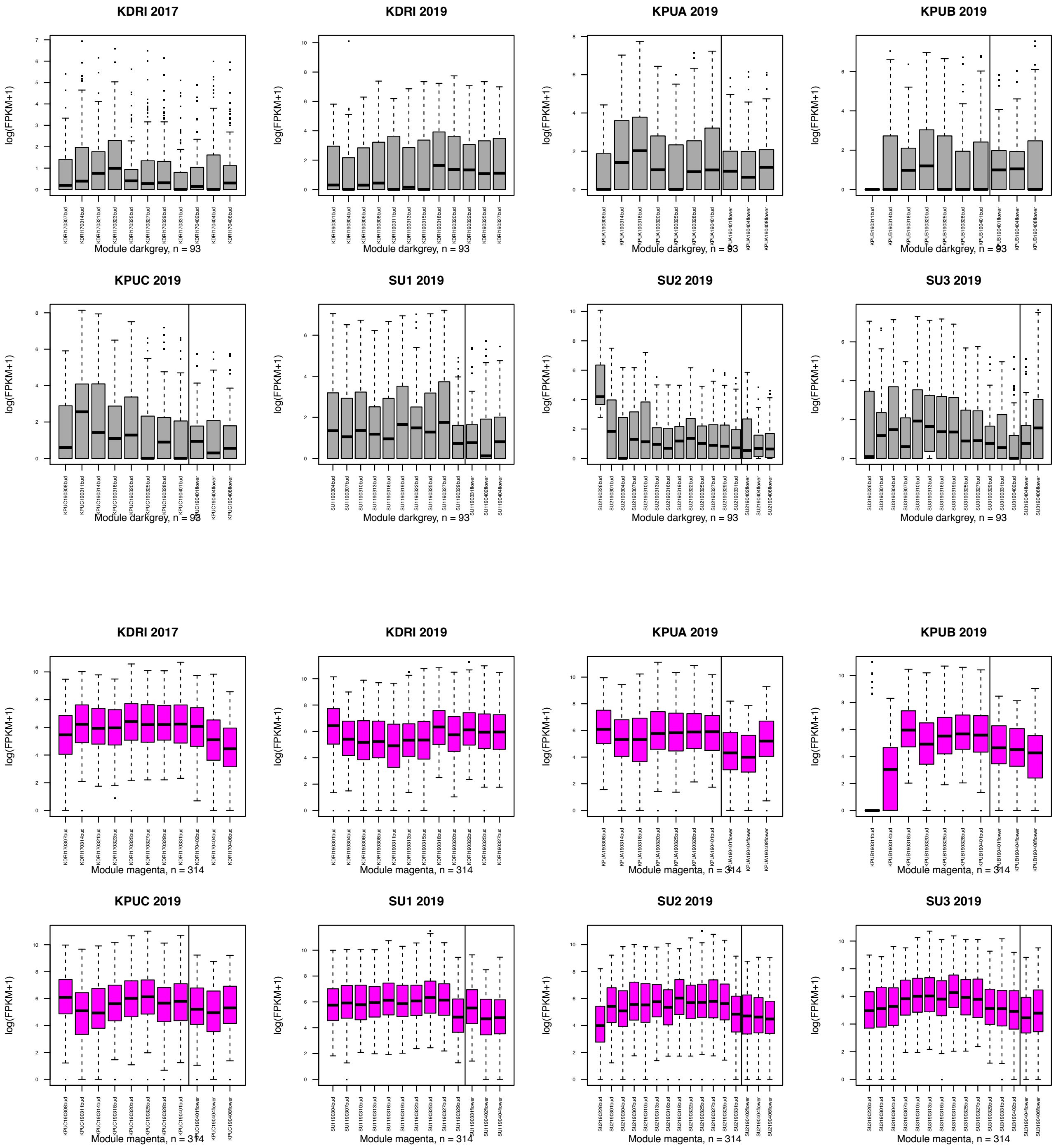

Supplementary Figure S2 (Continued.)

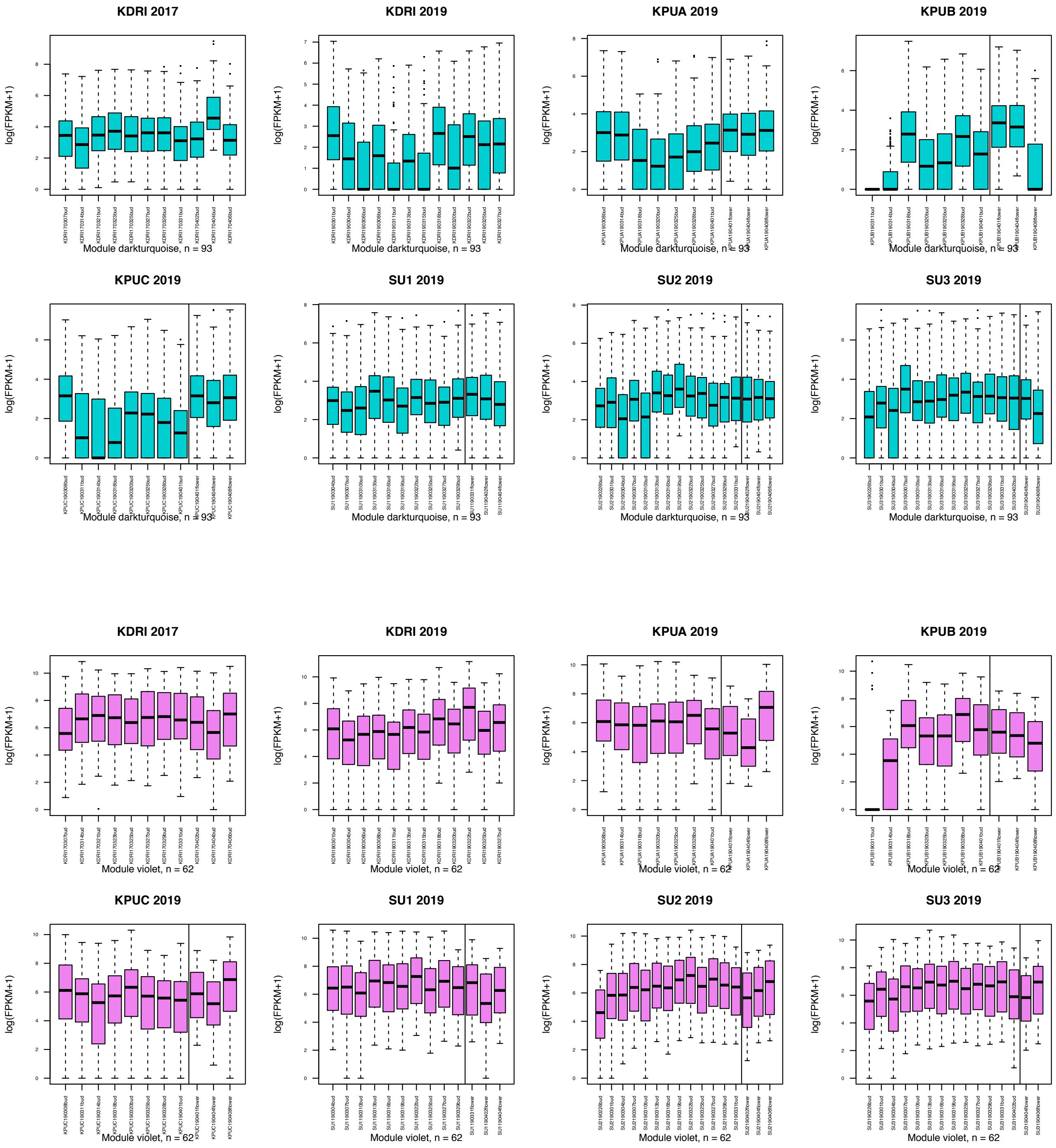

Supplementary Figure S2 (Continued.)

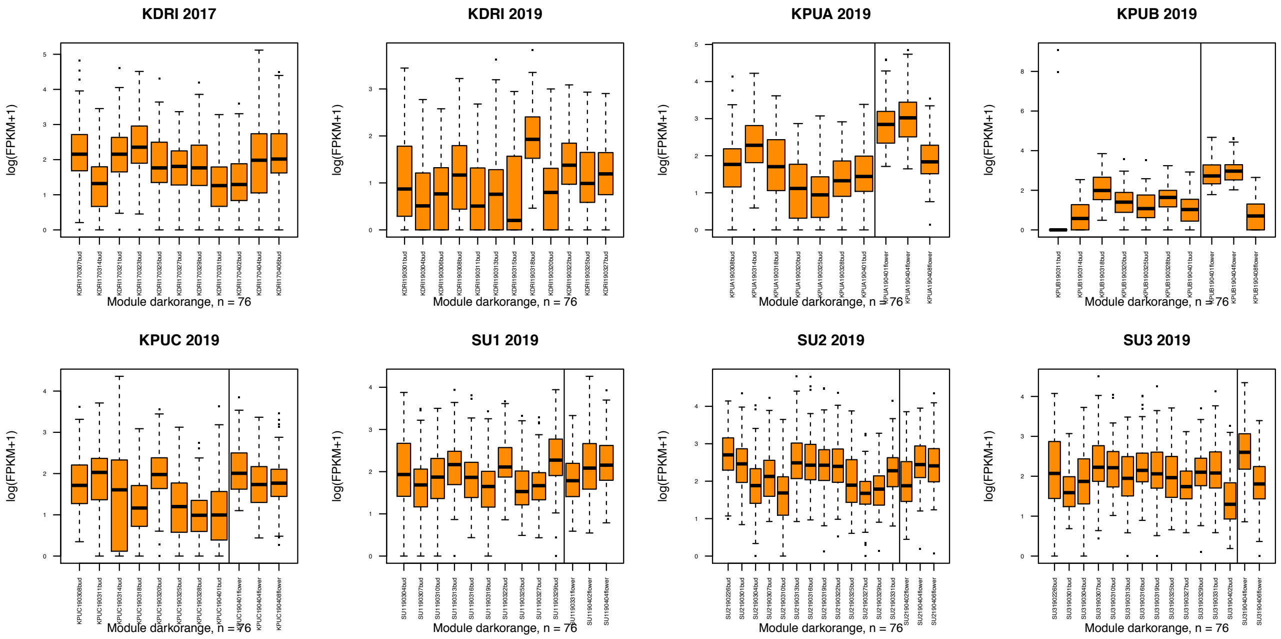

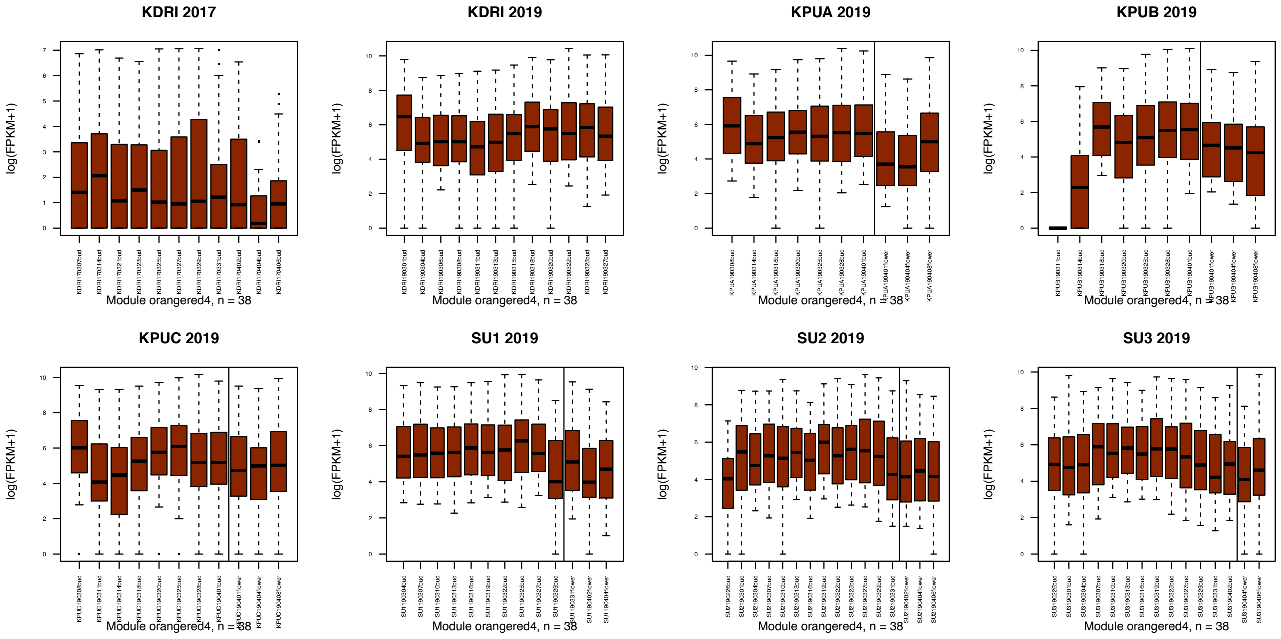

Supplementary Figure S2 (Continued.)

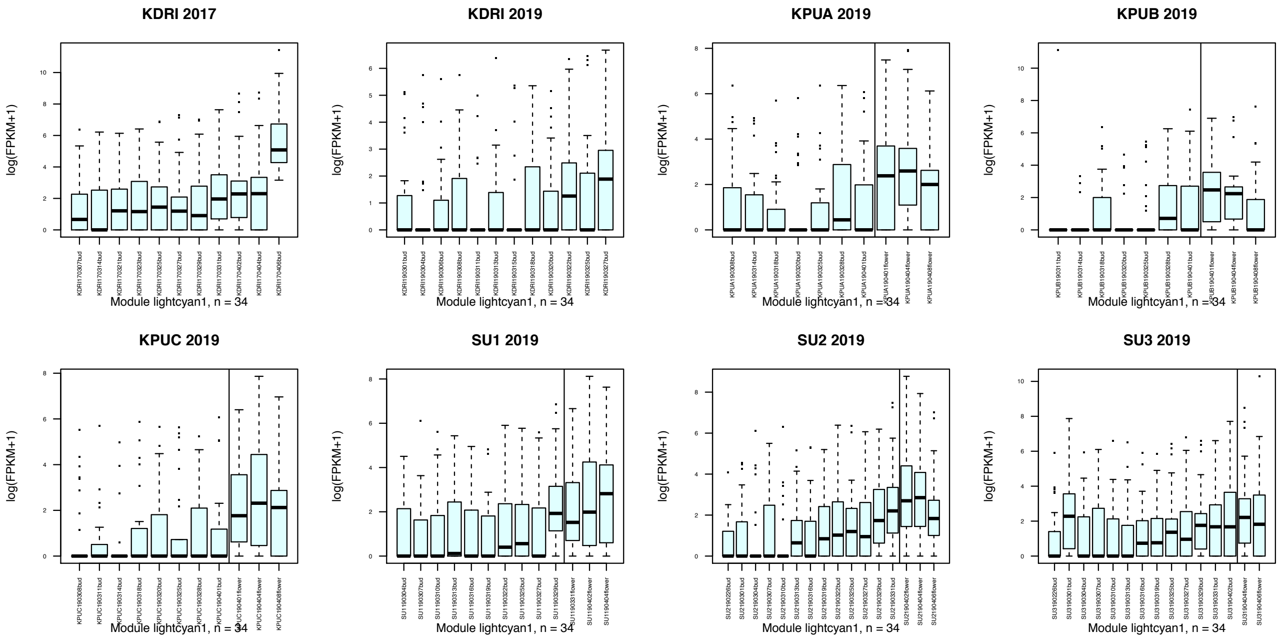

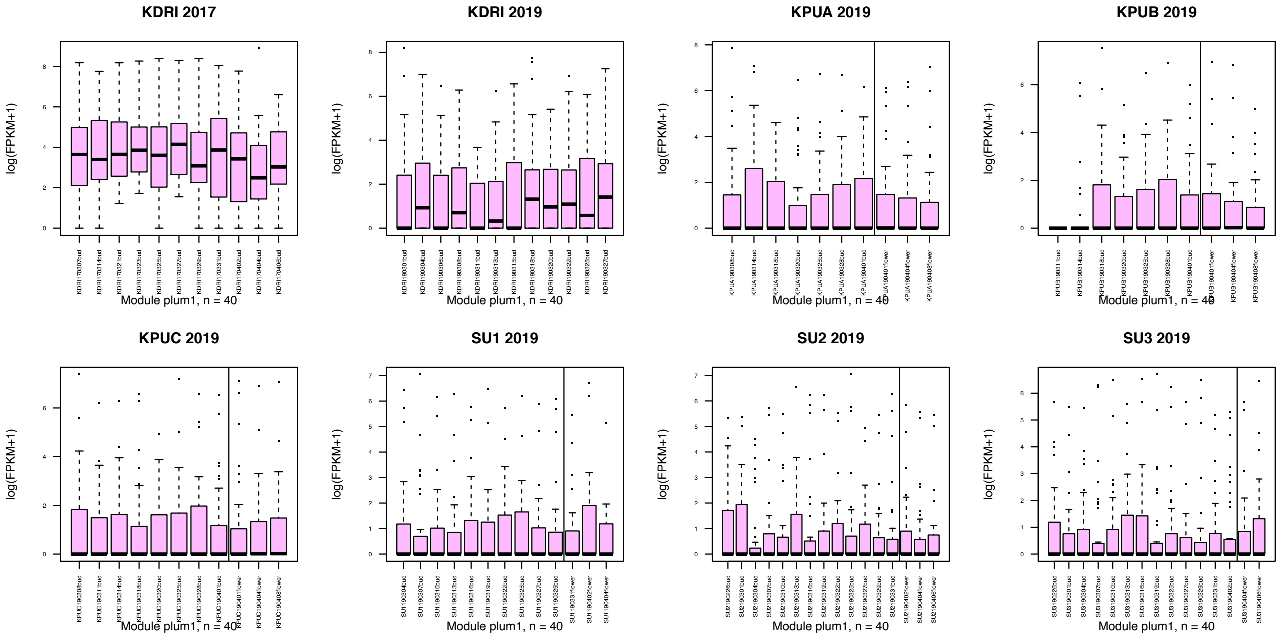

Supplementary Figure S2 (Continued.)

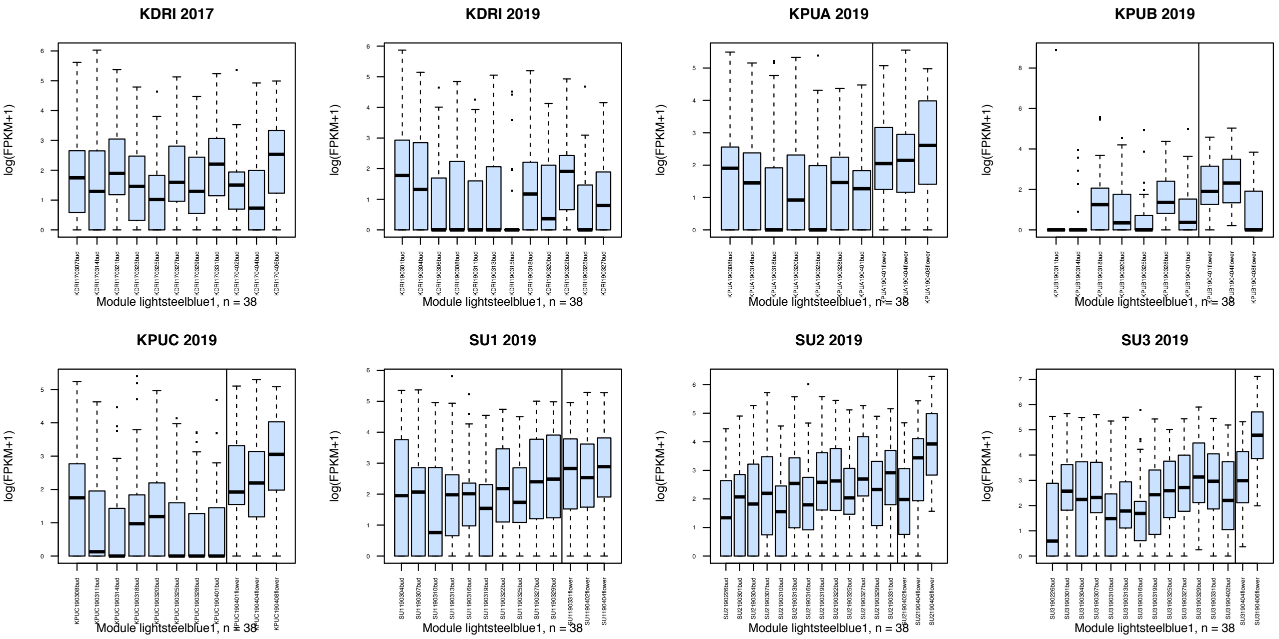

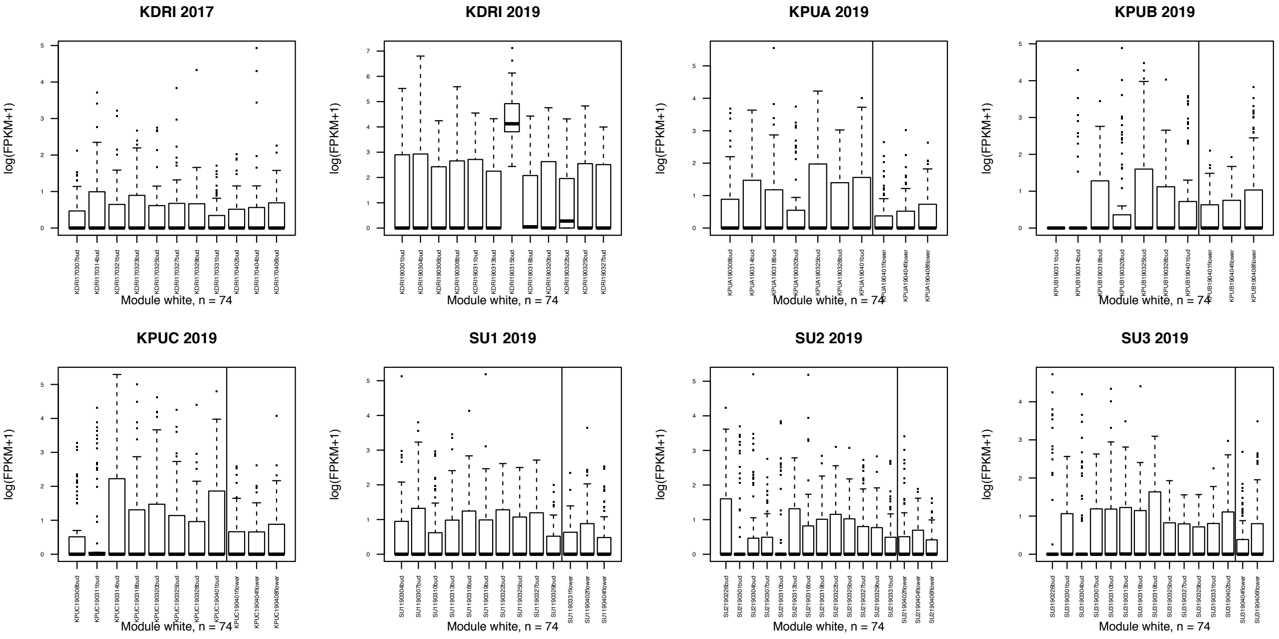

Supplementary Figure S2 (Continued.)

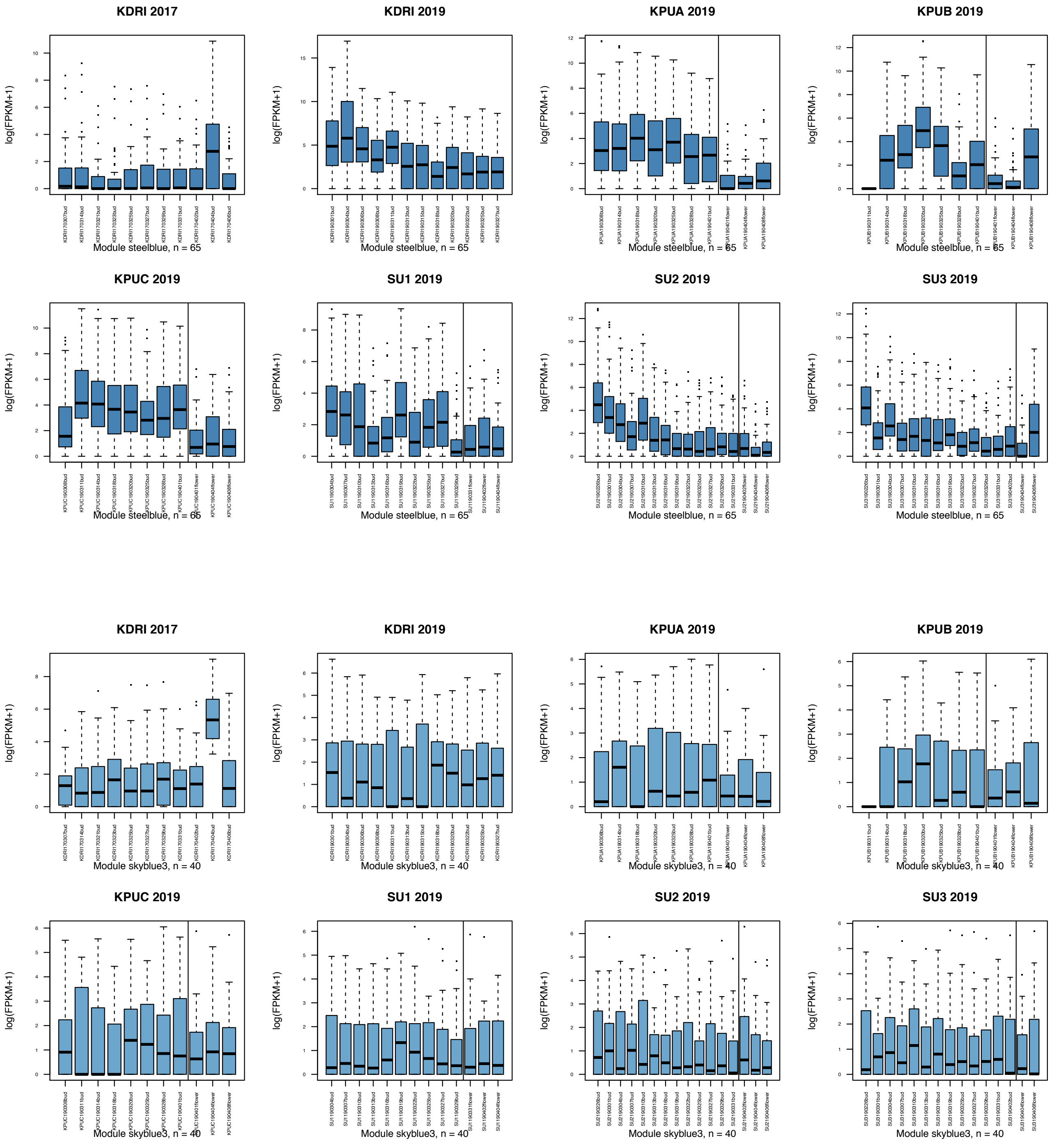

Supplementary Figure S2 (Continued.)

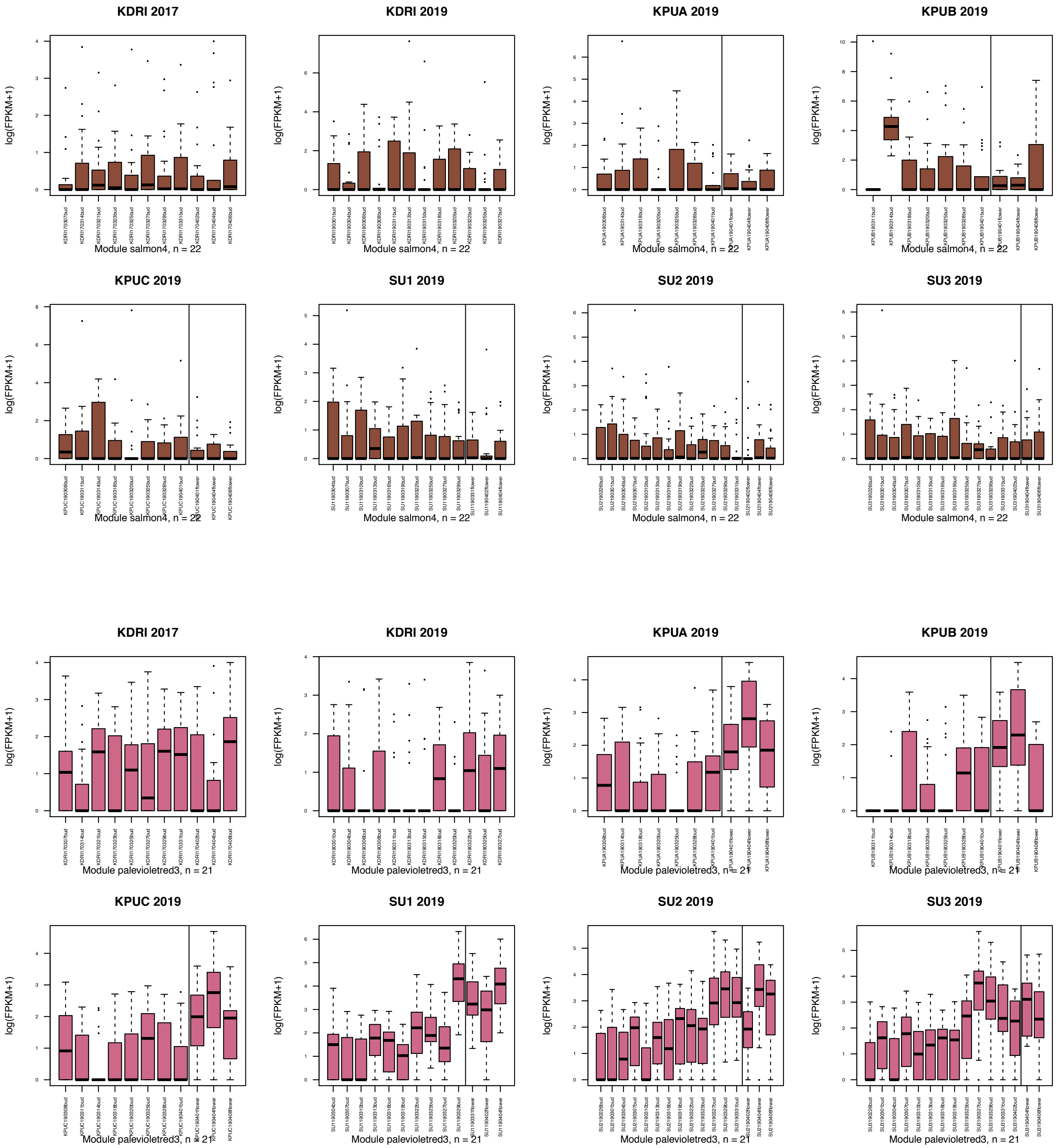

Supplementary Figure S2 (Continued.)

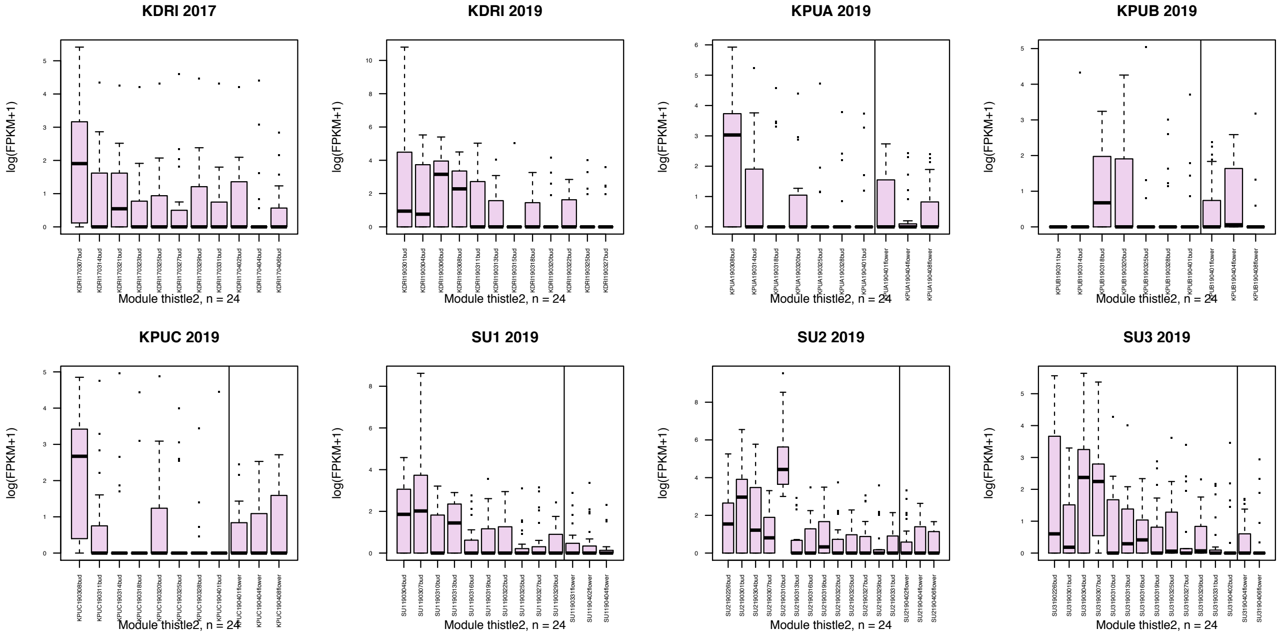

C

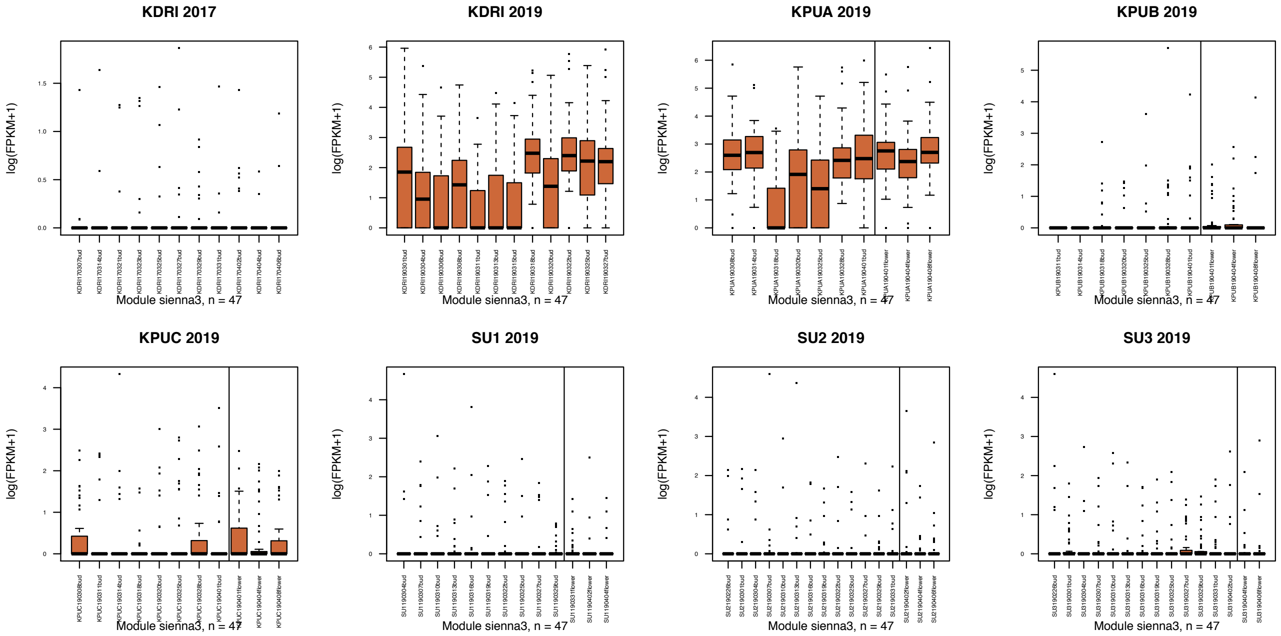

Supplementary Figure S2 (Continued.)

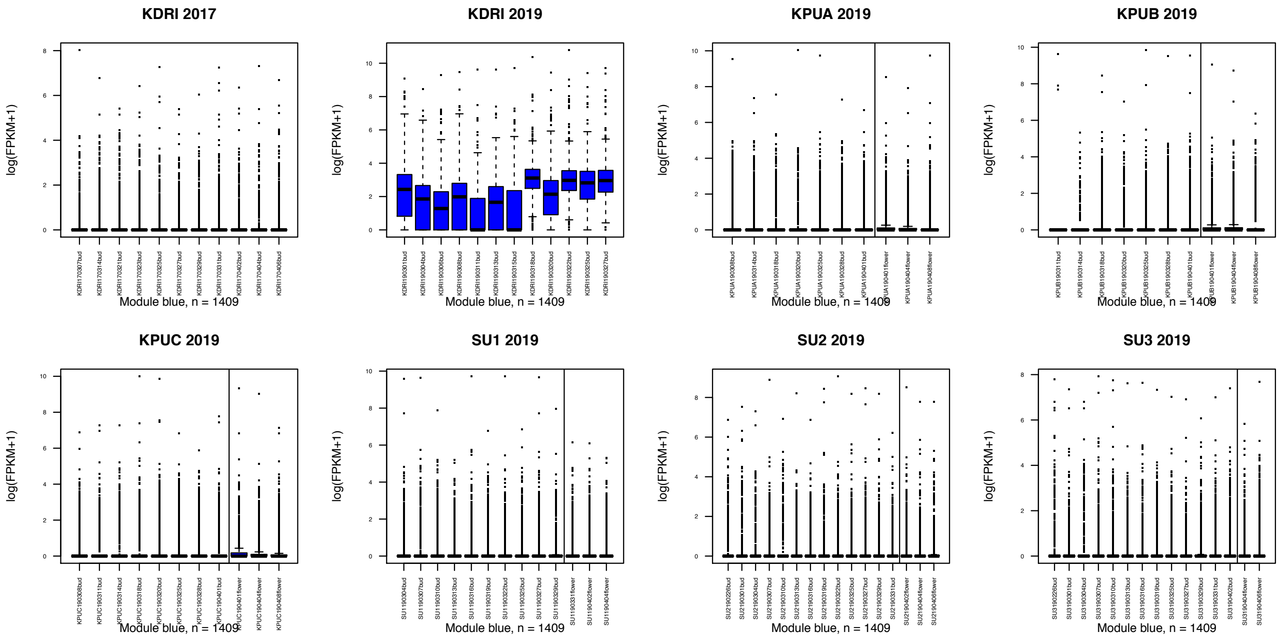

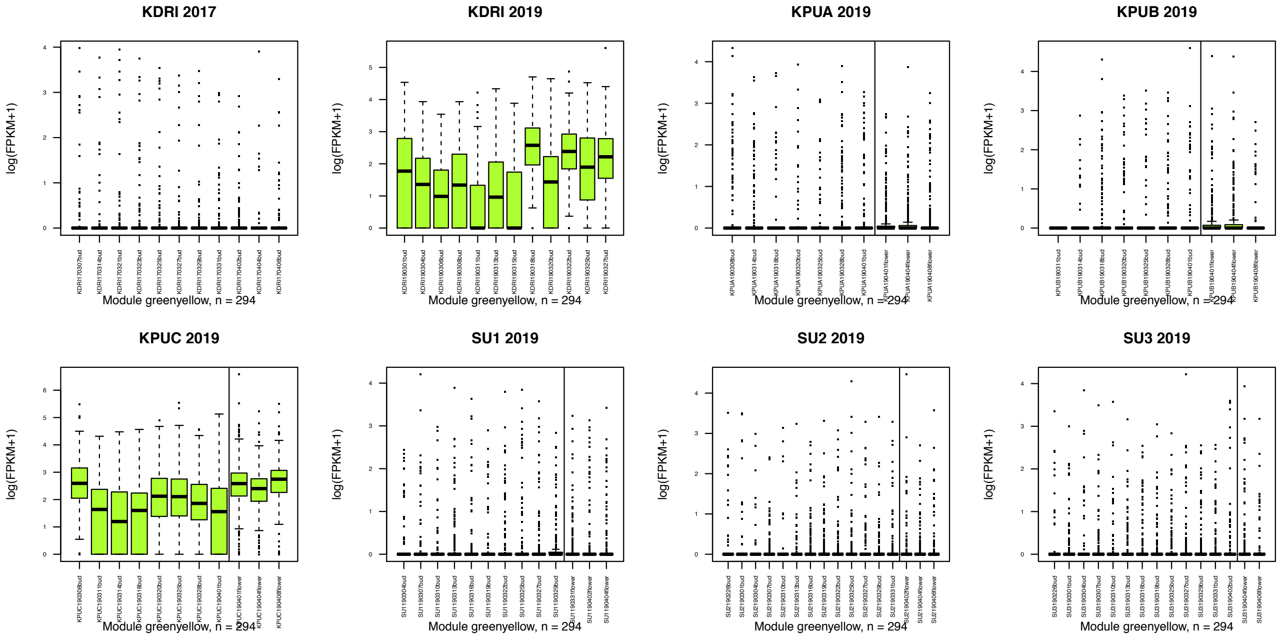

Supplementary Figure S2 (Continued.)

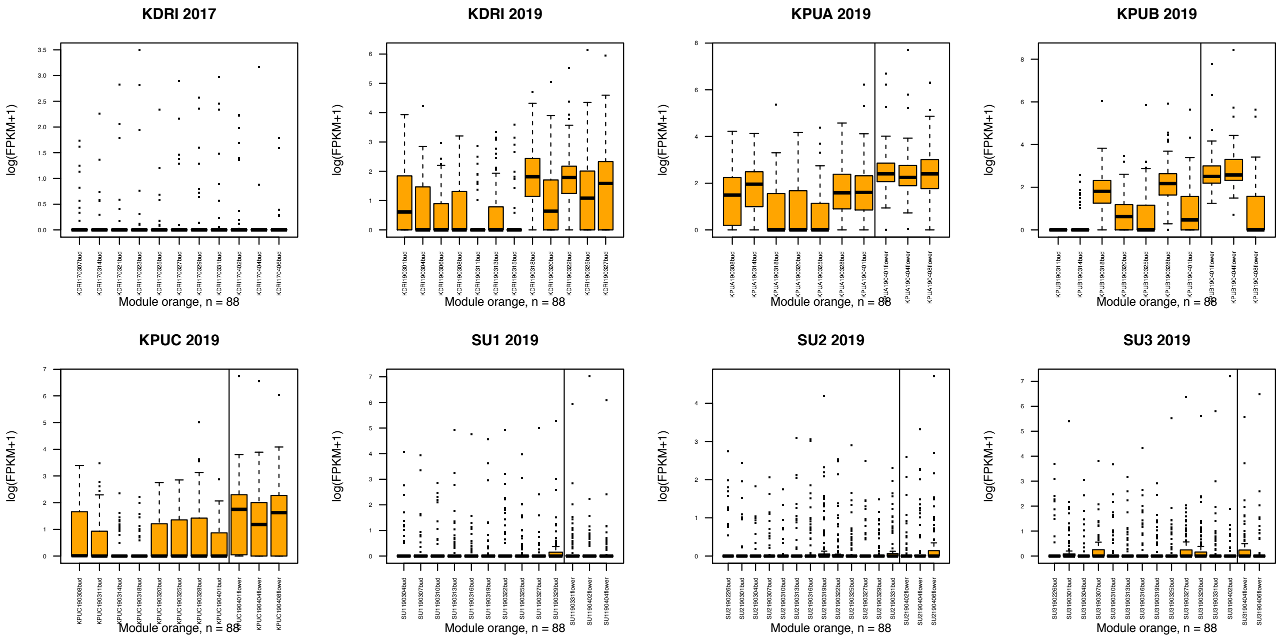

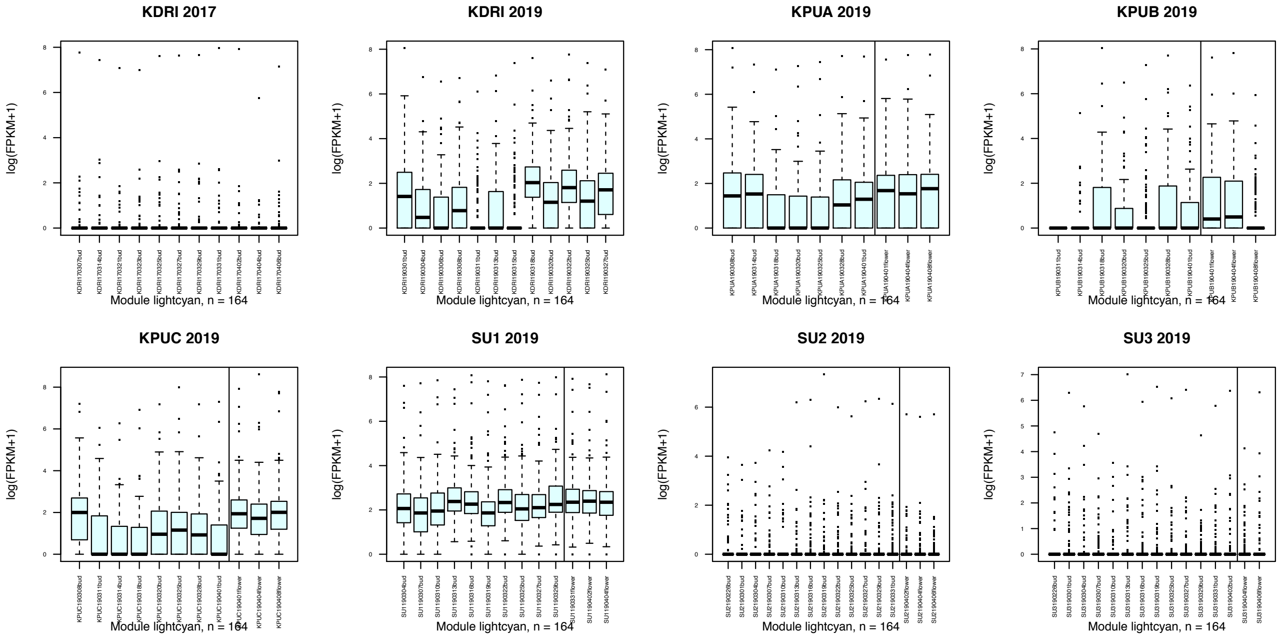

Supplementary Figure S2 (Continued.)

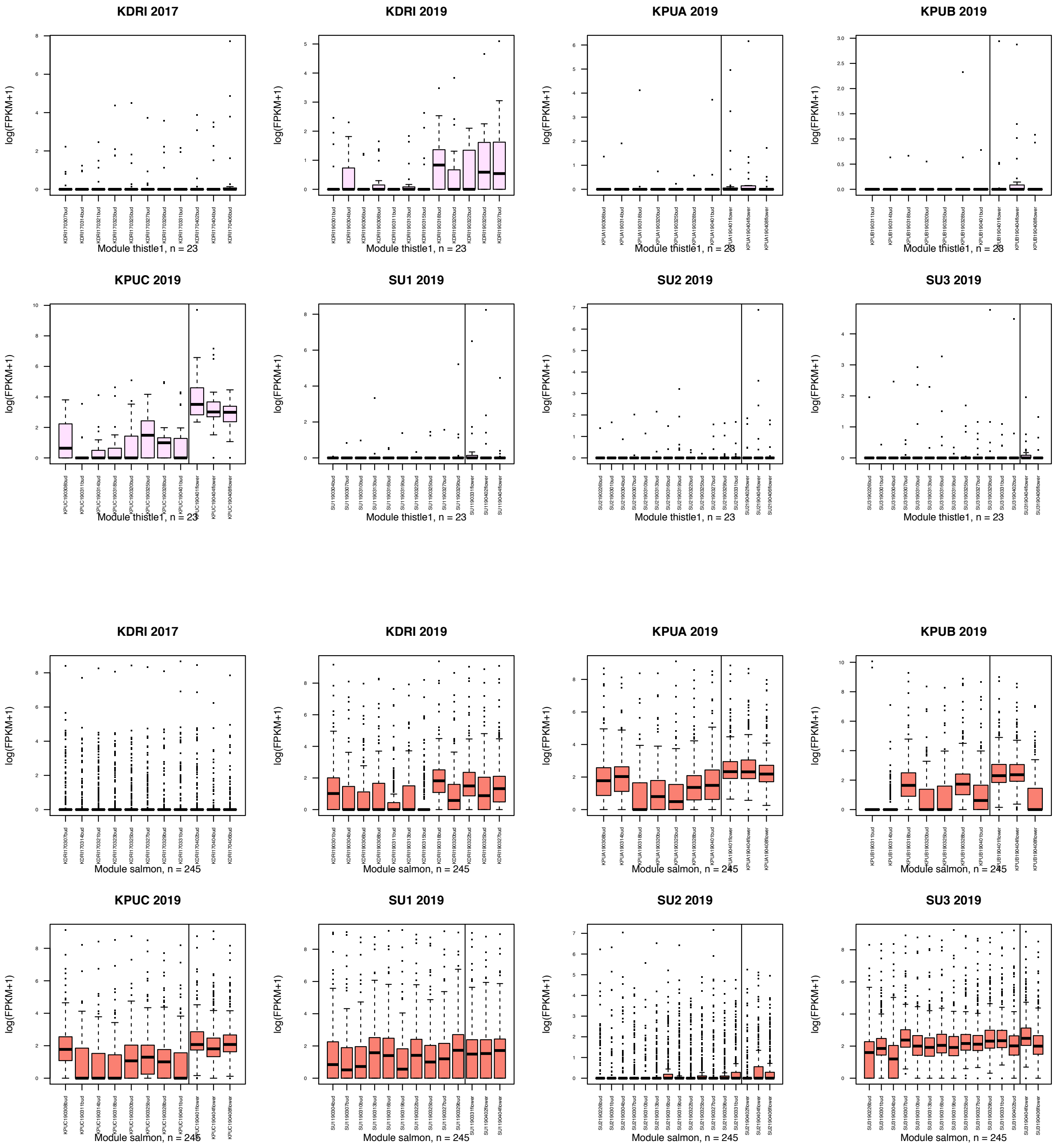

Supplementary Figure S2 (Continued.)

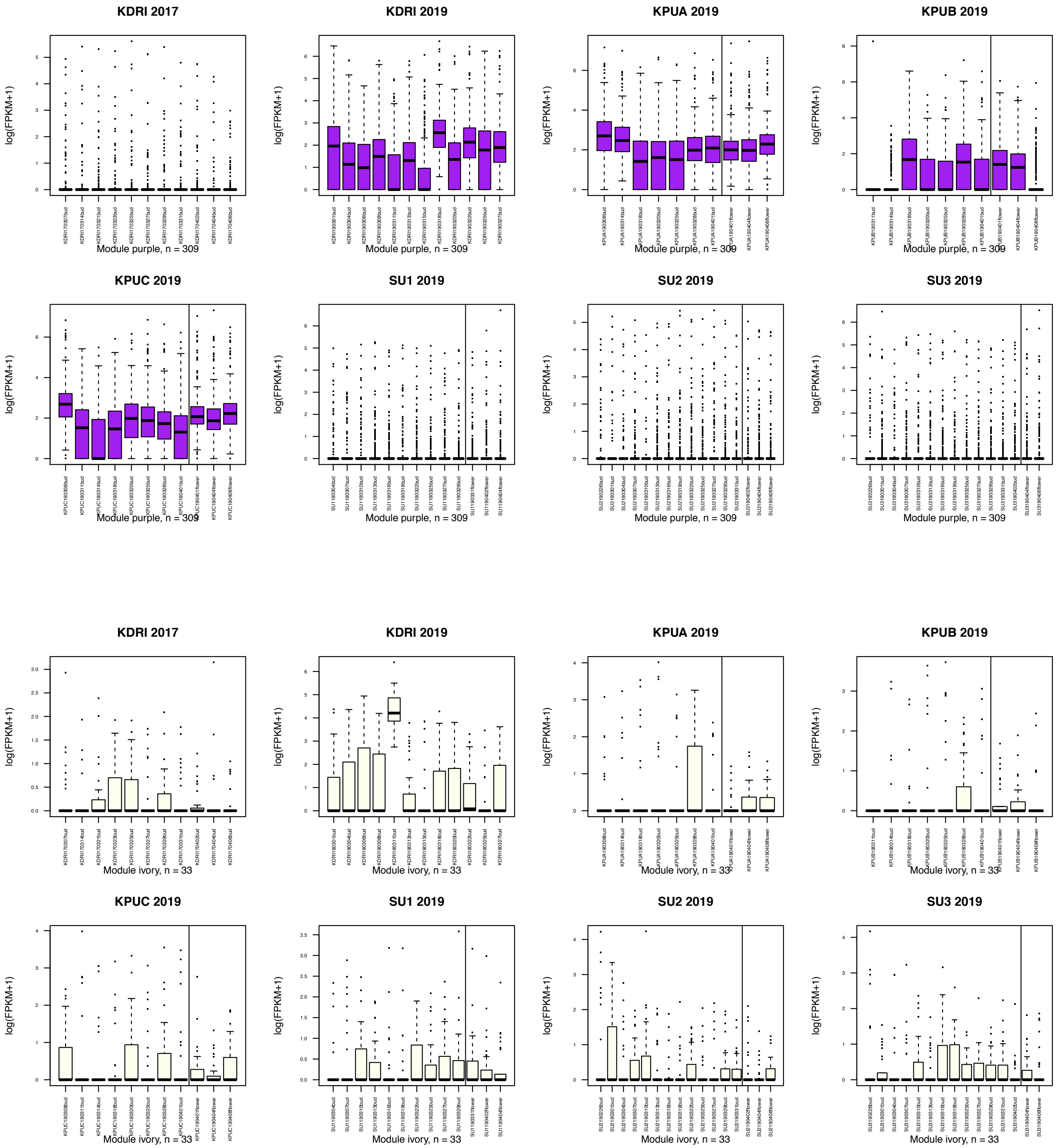

Supplementary Figure S2 (Continued.)

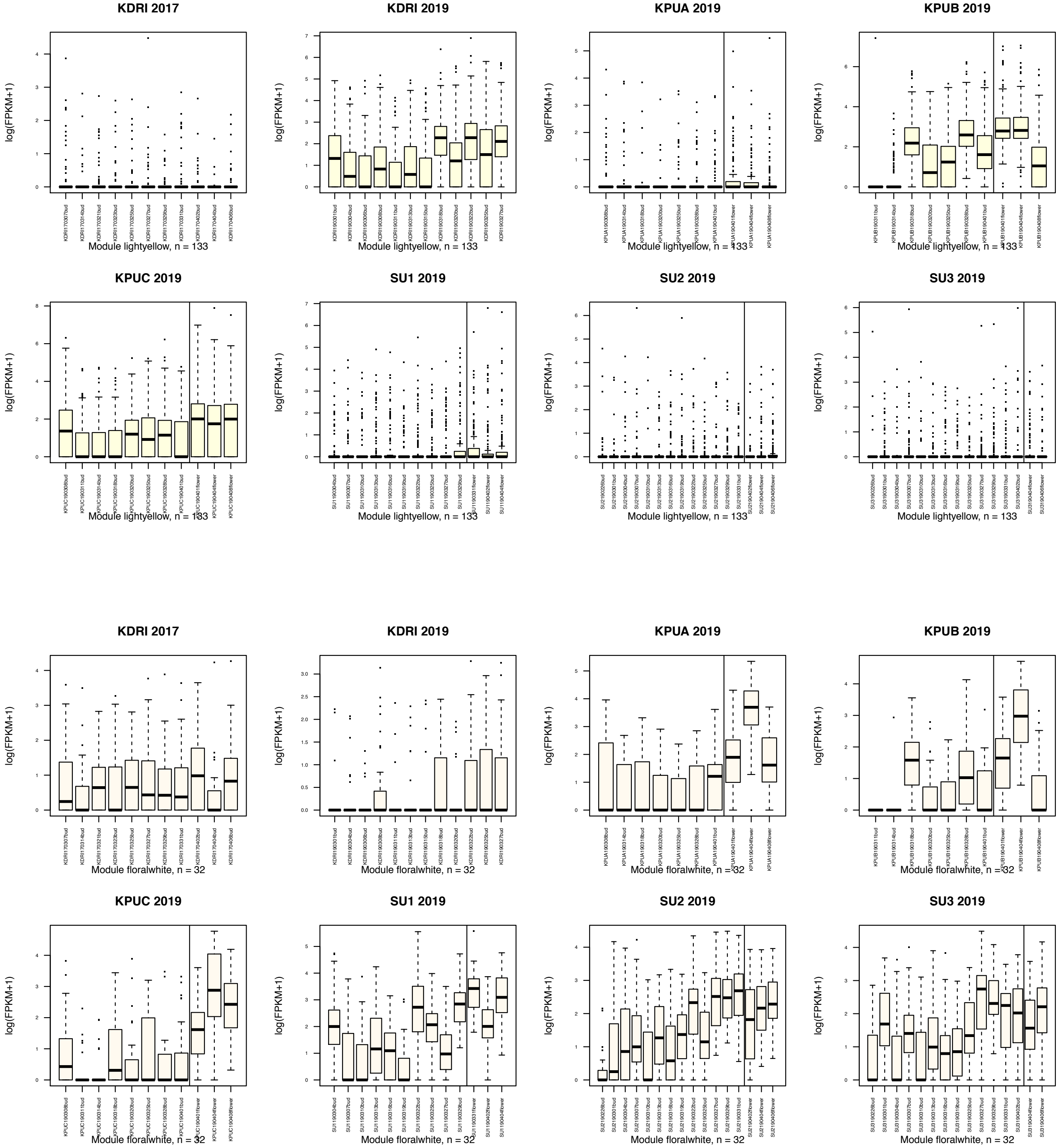

Supplementary Figure S2 (Continued.)

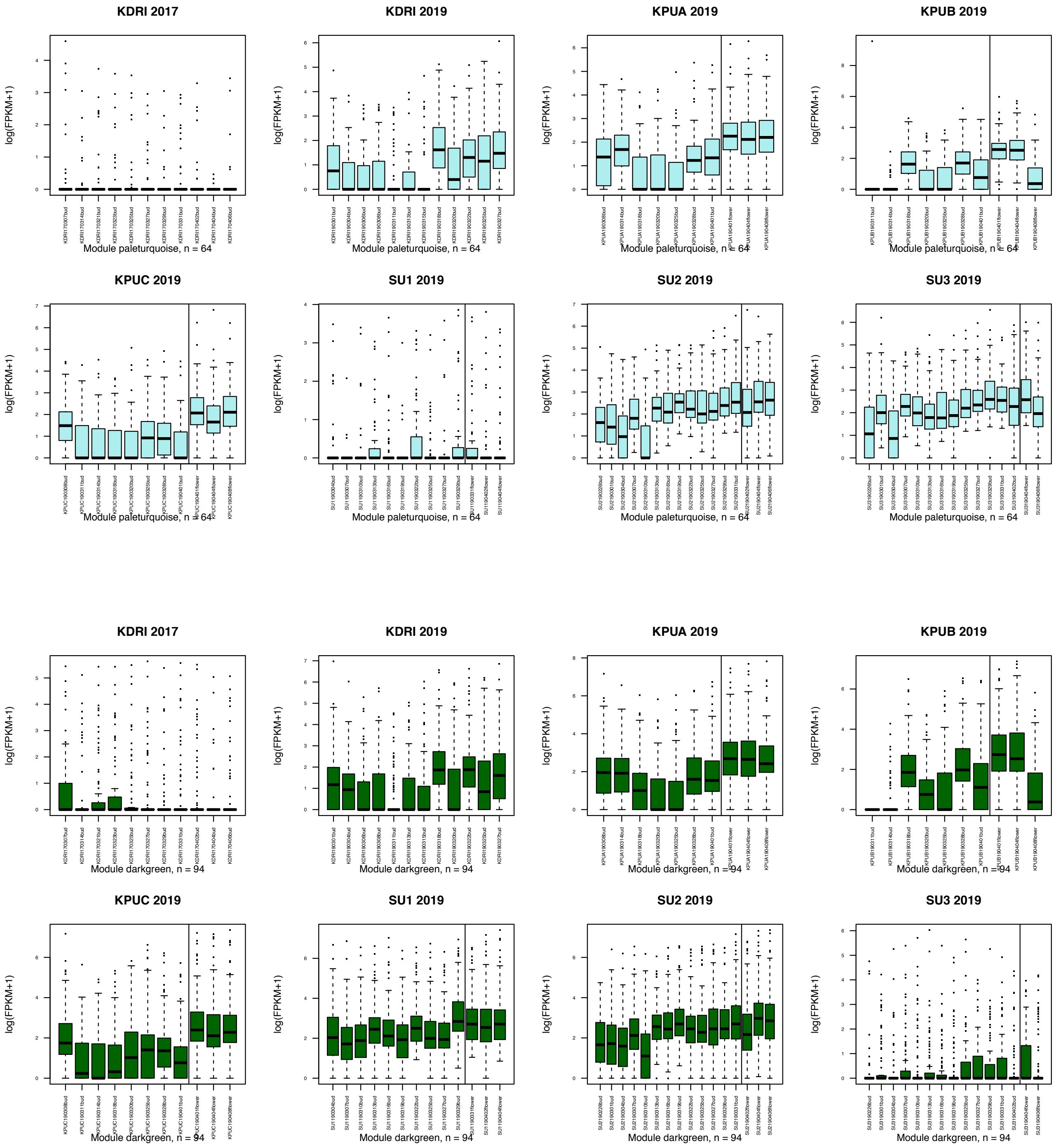

Supplementary Figure S2 (Continued.)

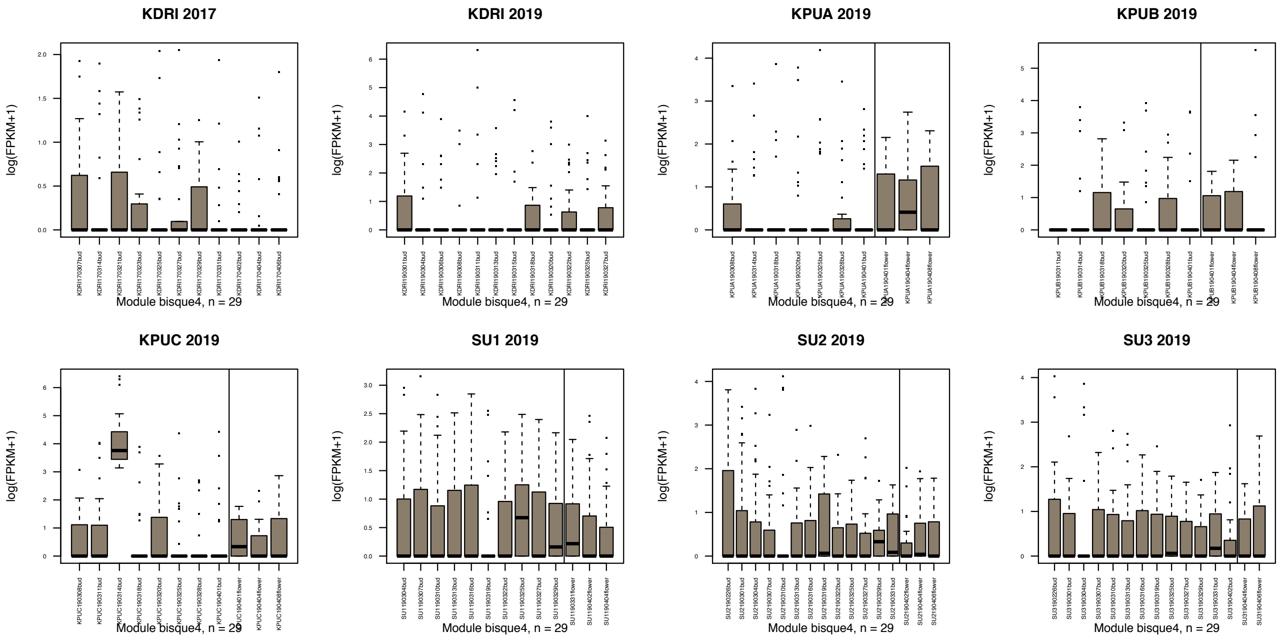

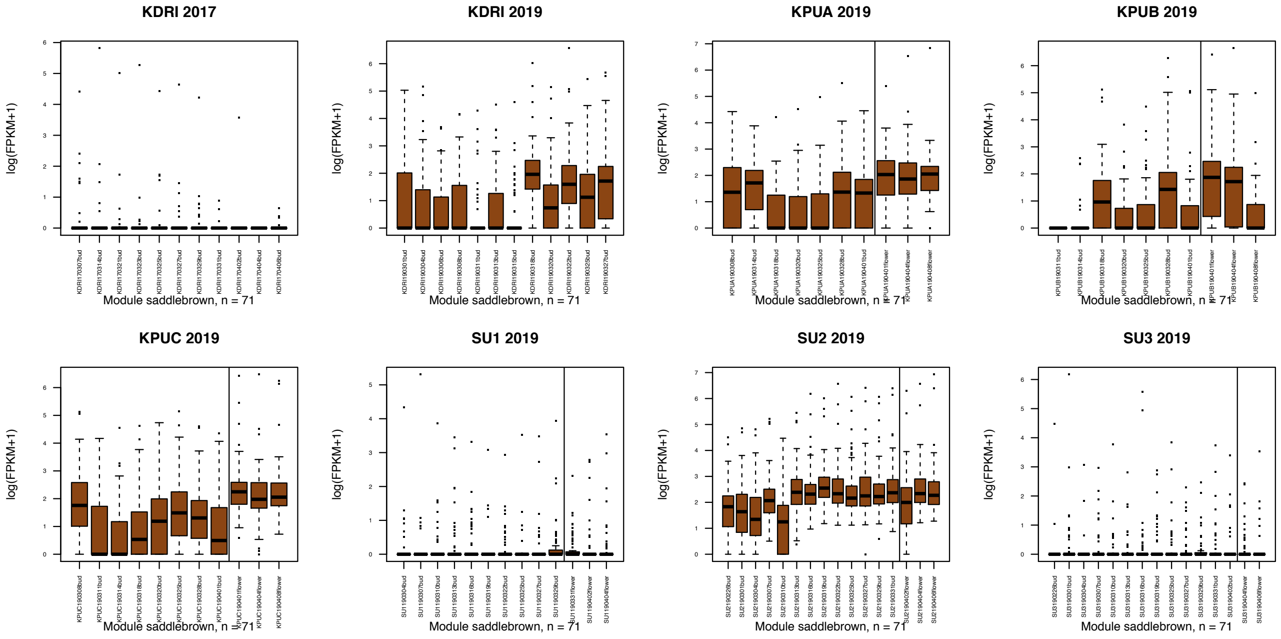

Supplementary Figure S2 (Continued.)

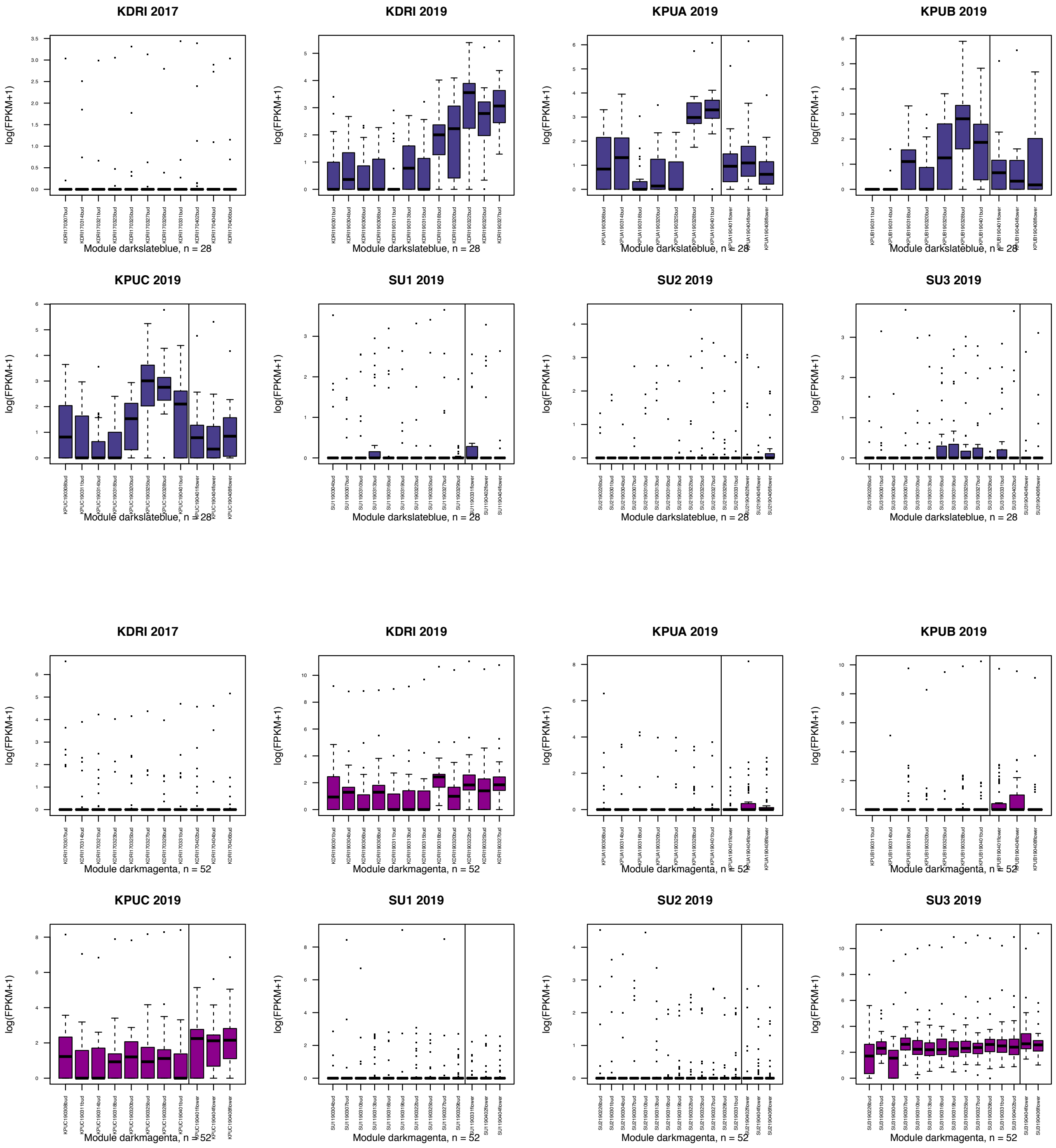

Supplementary Figure S2 (Continued.)

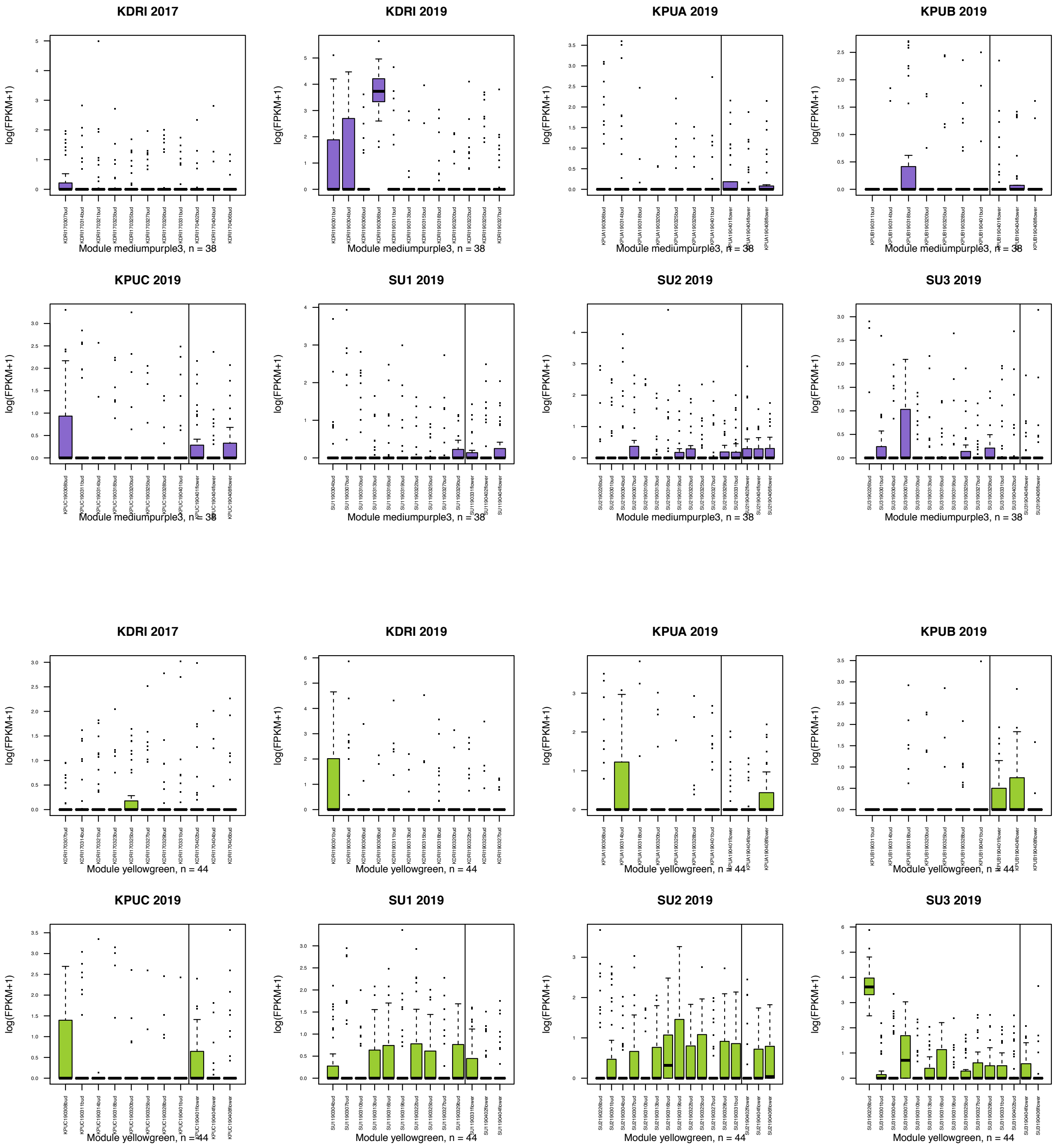

Supplementary Figure S2 (Continued.)

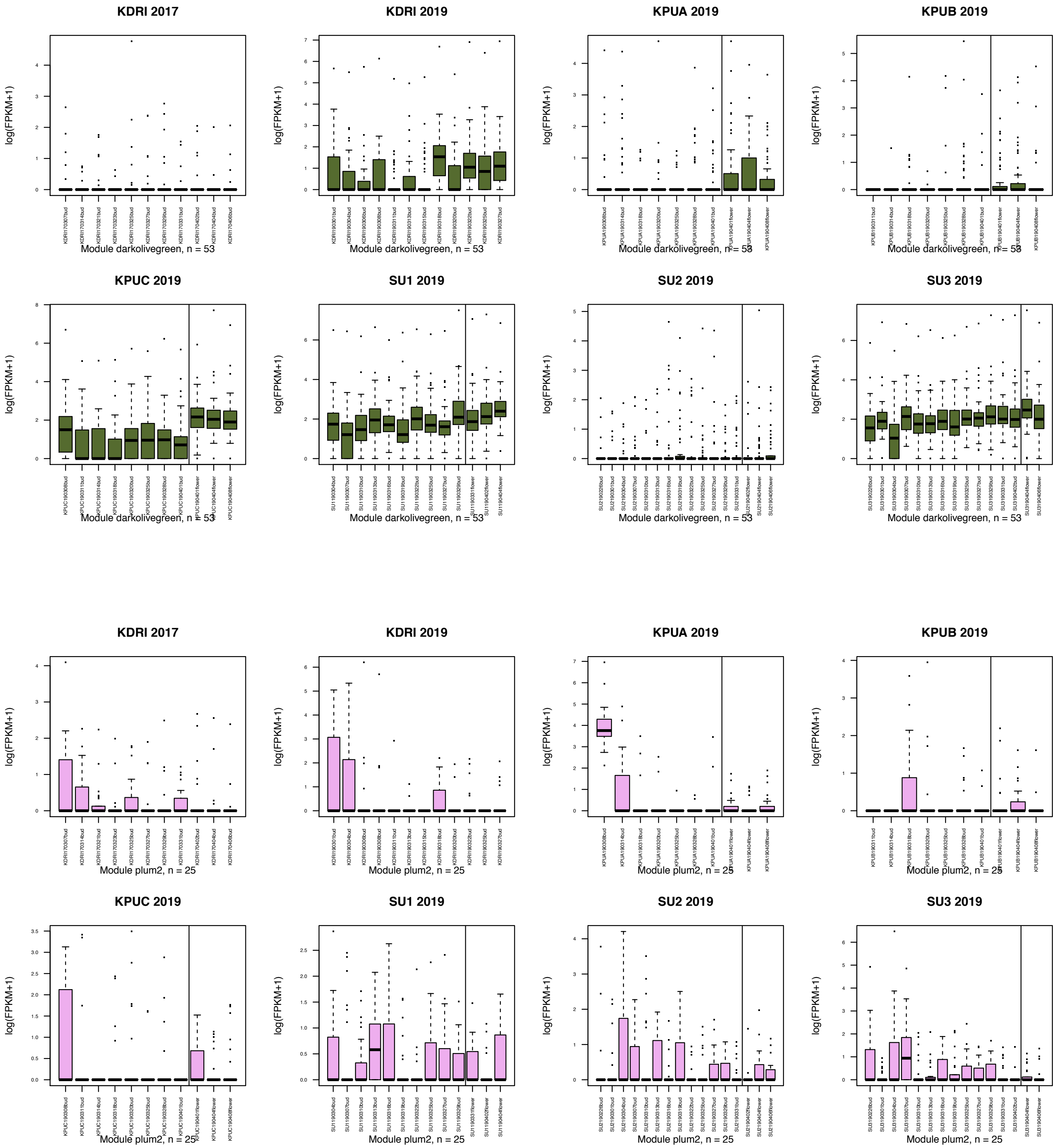

Supplementary Figure S2 (Continued.)

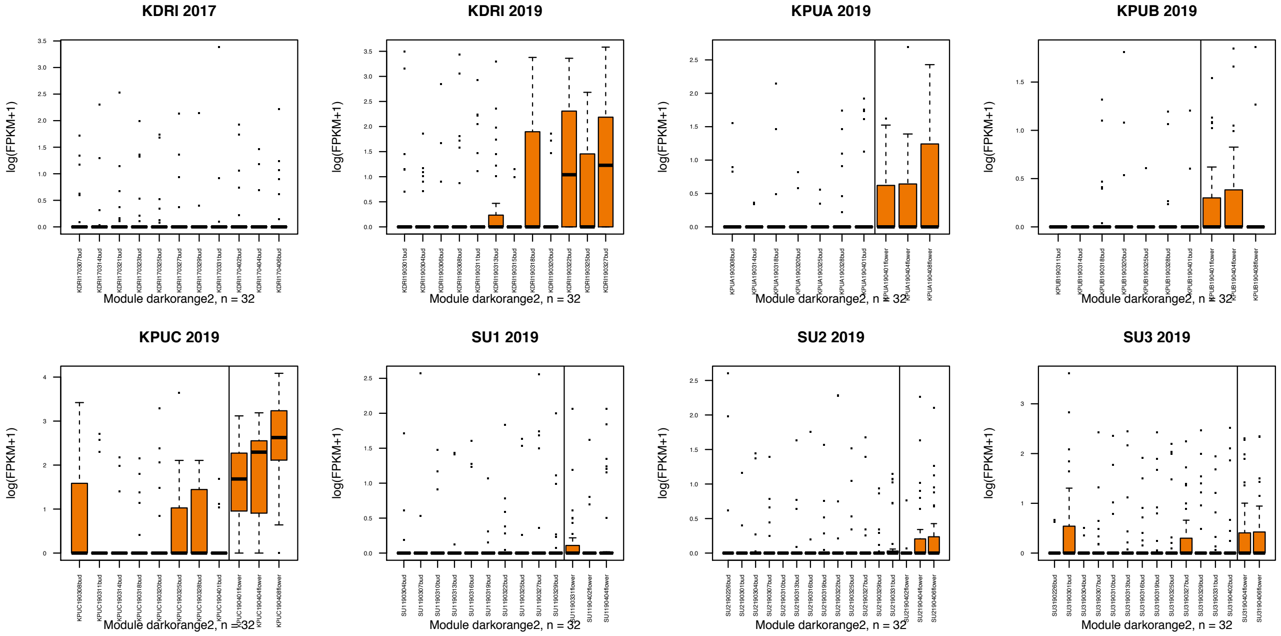

D

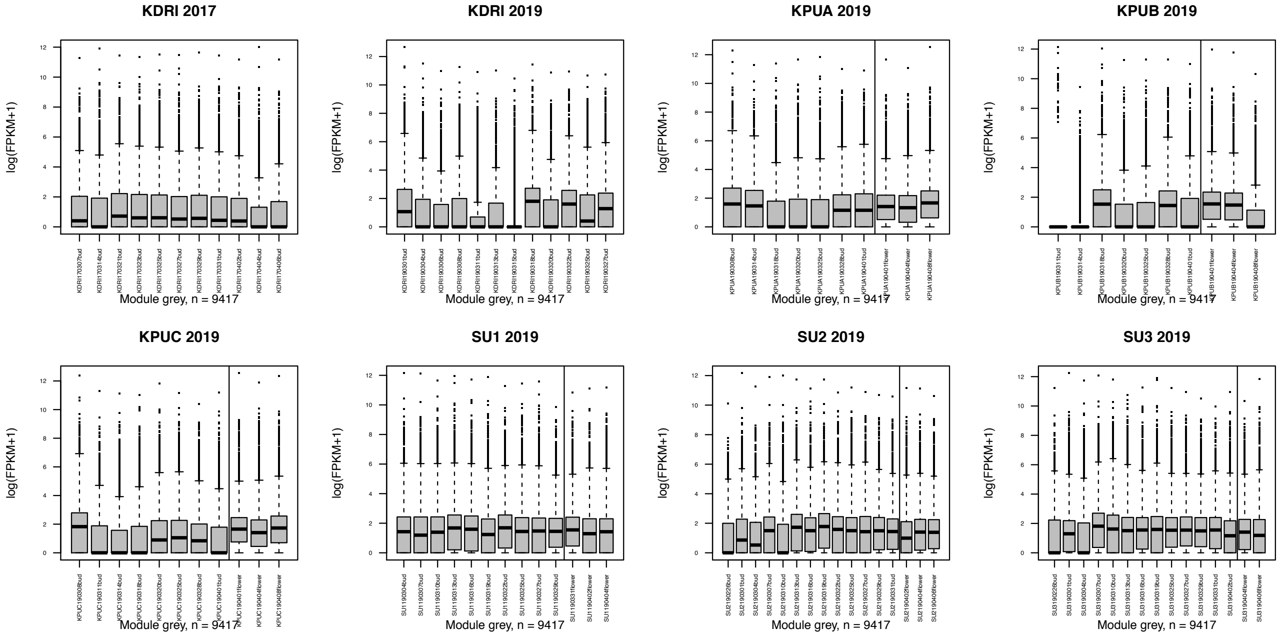

Supplementary Figure S2 (Continued.)

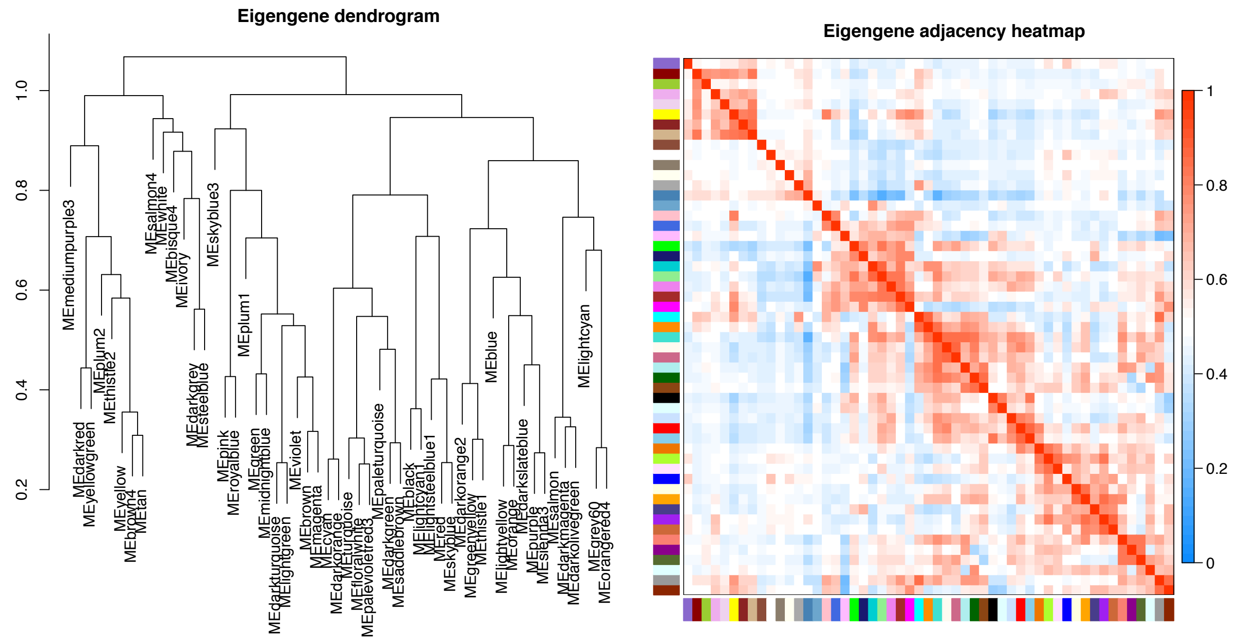

Supplementary Figure S3 Dendrogram and heatmap of gene modules represented by eigengenes obtained by WGCNA.

GO terms enriched in the module "darkred" (4-5 WBF)

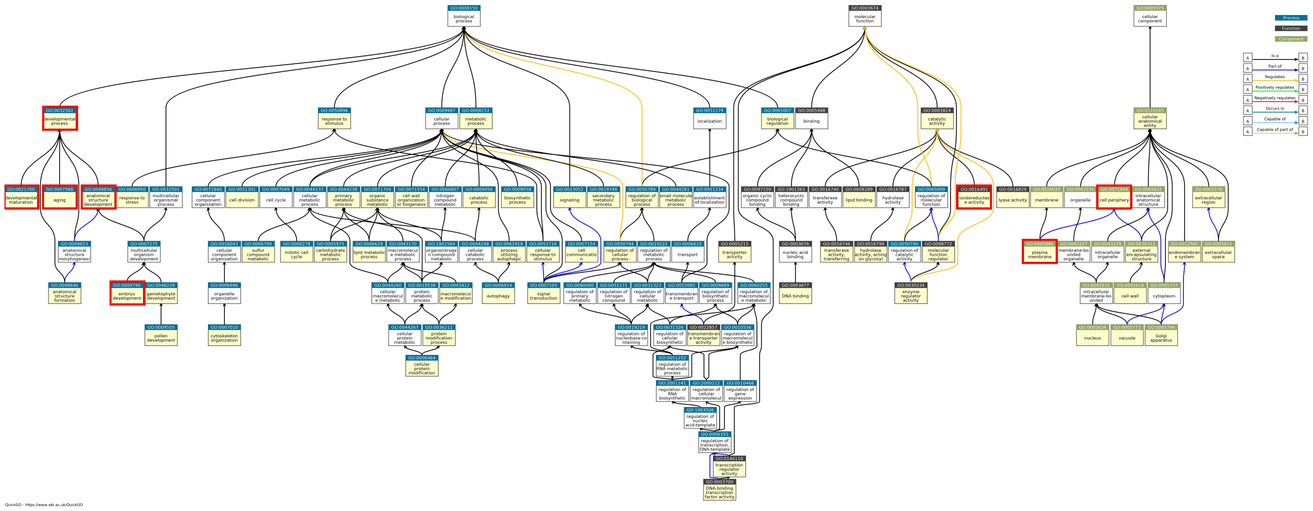

GO terms enriched in the module "tan " (4 WBF)

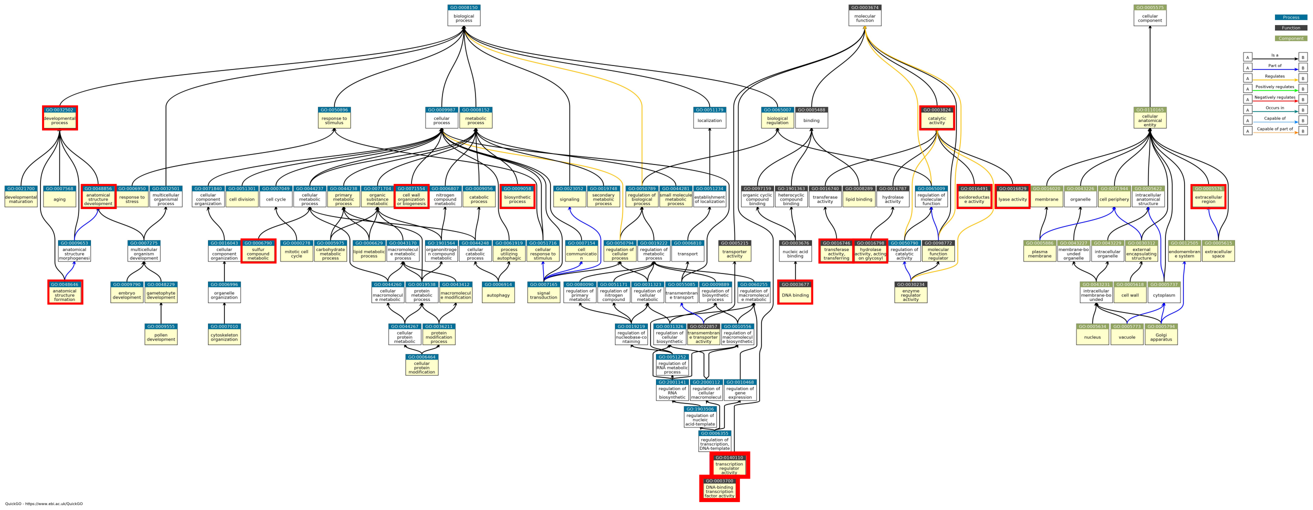

Supplementary Figure S4 Gene Ontology (GO) hierarchy trees. Yellow boxes and red frames indicate GO terms enriched in all seven modules and in each individual module, respectively.

GO terms enriched in the module “pink” (3 WBF)

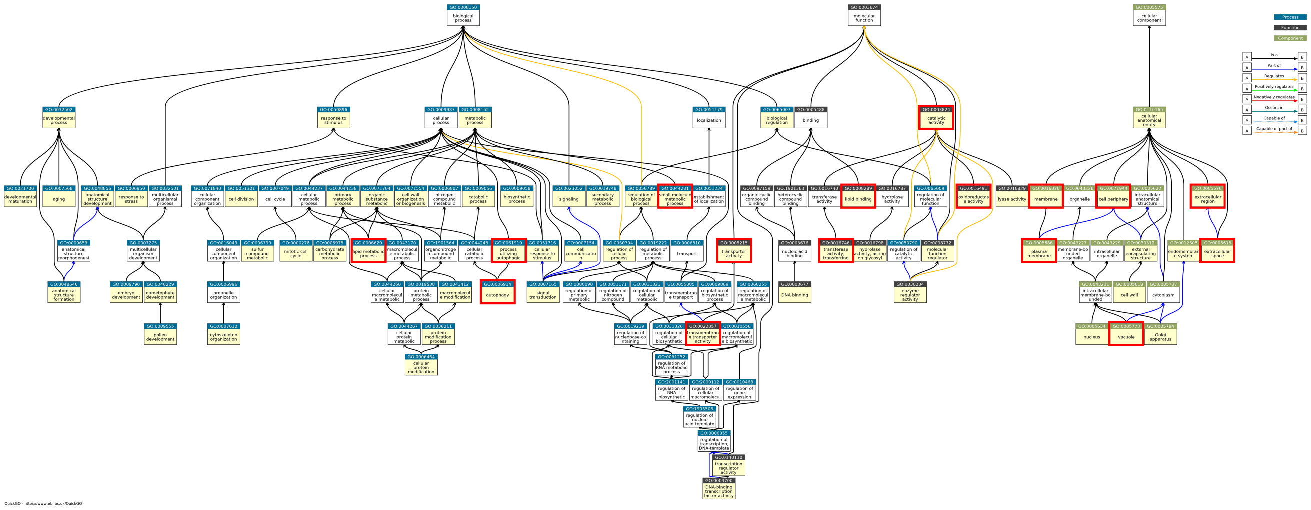

GO terms enriched in the module “royalblue” (2-3 WBF)

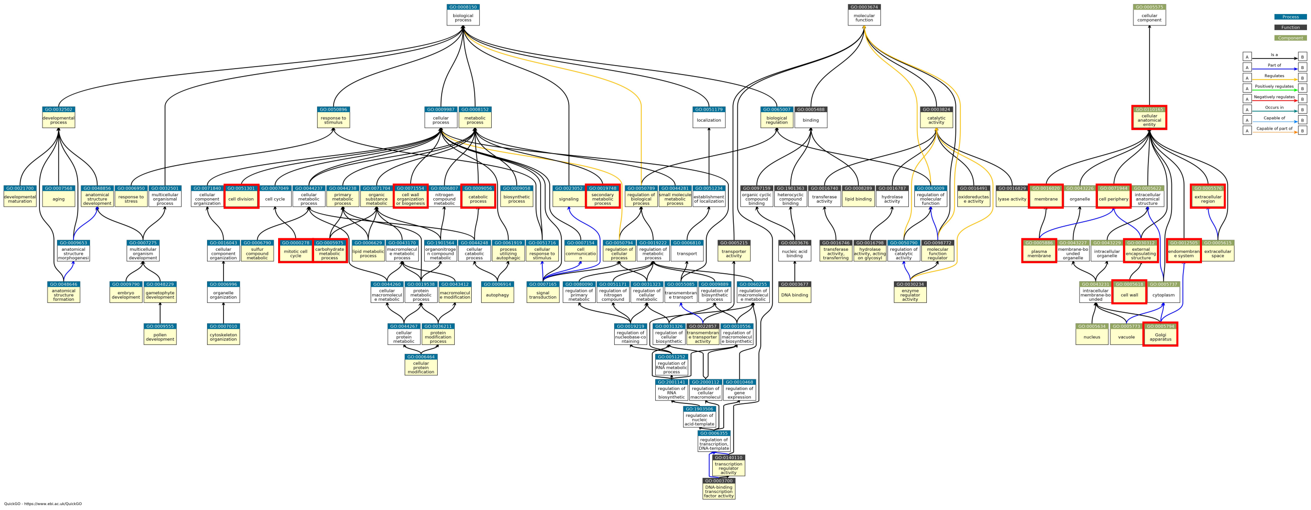

Supplementary Figure S4 (Continued.)

GO terms enriched in the module “midnightblue” (1-2 WBF)

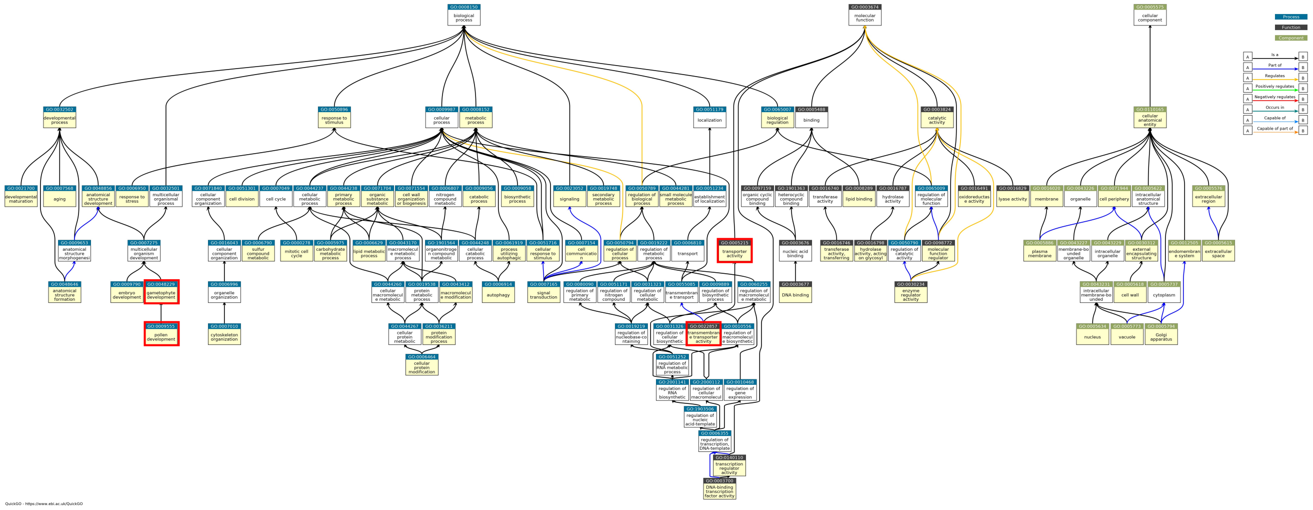

GO terms enriched in the module “black” (flowering day)

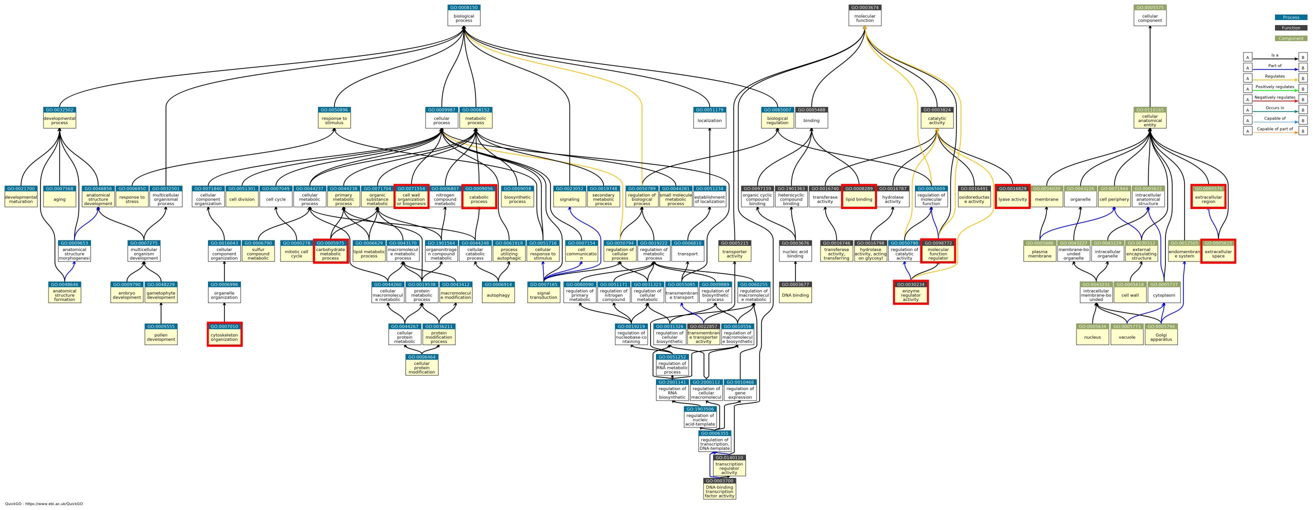

Supplementary Figure S4 (Continued.)

GO terms enriched in the module “skyblue” (1-2 WAF)

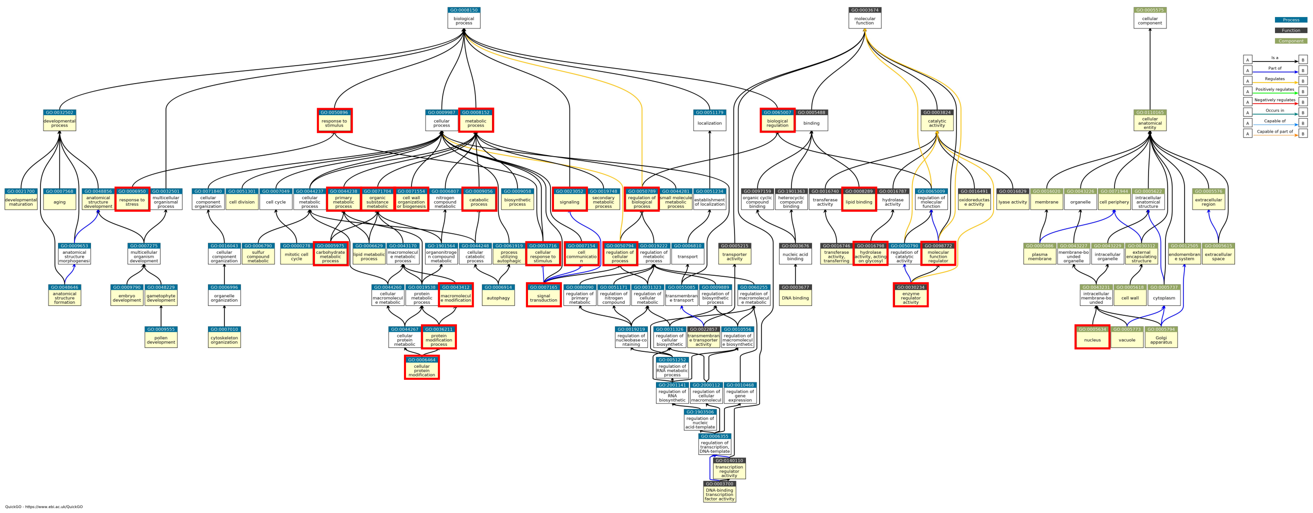

Supplementary Figure S4 (Continued.)
